# Perimenopausal and Menopausal Mammary Glands In A 4-Vinylcyclohexene Diepoxide Mouse Model

**DOI:** 10.1007/s10911-024-09569-x

**Published:** 2024-07-17

**Authors:** Kohei Saeki, Desiree Ha, Gregory Chang, Hitomi Mori, Ryohei Yoshitake, Xiwei Wu, Jinhui Wang, Yuan-Zhong Wang, Xiaoqiang Wang, Tony Tzeng, Hyun Jeong Shim, Susan L. Neuhausen, Shiuan Chen

**Affiliations:** 1 Department of Cancer Biology and Molecular Medicine, Beckman Research Institute of City of Hope, 1500 East Duarte Road, Duarte, CA 91010 USA; 2 Faculty of Veterinary Medicine, Okayama University of Science, Ehime, Japan; 3 Integrative Genomics Core, Beckman Research Institute of City of Hope, Duarte, CA USA; 4 Department of Population Sciences, Beckman Research Institute of City of Hope, Duarte, CA USA

**Keywords:** PBDEs, 4-Vinylcyclohexene Diepoxide (VCD), Windows of Susceptibility, Mammary Gland, Breast Cancer, Single-Cell RNA Sequencing

## Abstract

As both perimenopausal and menopausal periods are recognized critical windows of susceptibility for breast carcinogenesis, development of a physiologically relevant model has been warranted. The traditional ovariectomy model causes instant removal of the entire hormonal repertoire produced by the ovary, which does not accurately approximate human natural menopause with gradual transition. Here, we characterized the mammary glands of 4-vinylcyclohexene diepoxide (VCD)-treated animals at different time points, revealing that the model can provide the mammary glands with both perimenopausal and menopausal states. The perimenopausal gland showed moderate regression in ductal structure with no responsiveness to external hormones, while the menopausal gland showed severe regression with hypersensitivity to hormones. Leveraging the findings on the VCD model, effects of a major endocrine disruptor (polybrominated diphenyl ethers, PBDEs) on the mammary gland were examined during and after menopausal transition, with the two exposure modes; low-dose, chronic (environmental) and high-dose, subacute (experimental). All conditions of PBDE exposure did not augment or compromise the macroscopic ductal reorganization resulting from menopausal transition and/or hormonal treatments. Single-cell RNA sequencing revealed that the experimental PBDE exposure during the post-menopausal period caused specific transcriptomic changes in the non-epithelial compartment such as *Errfi1* upregulation in fibroblasts. The environmental PBDE exposure resulted in similar transcriptomic changes to a lesser extent. In summary, the VCD mouse model provides both perimenopausal and menopausal windows of susceptibility for the breast cancer research community. PBDEs, including all tested models, may affect the post-menopausal gland including impacts on the non-epithelial compartments.

**Supplementary Information:**

The online version contains supplementary material available at 10.1007/s10911-024-09569-x.

## Introduction

During menopausal transition, identified as a window of susceptibility (WOS), the mammary gland becomes hypersensitive to fluctuating sex hormones and the presence of external hormone mimics, increasing the risk for diseases such as breast cancer. Perimenopause is a delicate interval in which the mammary gland gradually undergoes complex reorganization. During this period, female reproductive organs and mammary glands gradually change their physiology in response to fluctuating endogenous hormone levels [1–3]. At menopause, the near complete elimination of circulating levels of 17β-estradiol (E2) and progesterone (P4) further drives mammary gland regression as well as starvation to these hormones, which put the mammary glands at risk for exposure to exogenous sex hormones and endocrine-disrupting chemicals (EDCs) [1–3].

Although EDCs have been linked to carcinogenesis risk, their exact effects are still being defined experimentally. One class of EDCs are polybrominated diphenyl ethers (PBDEs), pollutants introduced in the 1970s that were frequently used as household flame retardants for furniture, plastics, building materials, and electrical equipment [4]. Although the use of PBDEs was gradually discontinued due to their hazardous health effects and implications with carcinogenesis since 2004, massive repositories of PBDEs are still present through electronic waste recycling [5]. A case–control study quantifying 14 PBDE congeners in adipose tissues of women with and without breast cancer concluded that women exposed to PBDEs were associated with a higher risk of breast cancer development [6]. Despite their commercial phase out, PBDEs continue to persist in the environment due to their high lipophilicity, stability, and long half-lives in human tissues [7]. This prevalence of PBDEs has detrimental impacts on women’s health, potentially by augmenting estrogen-facilitated gene expression and modulating immune regulation [2, 8]. Many studies have attempted to demonstrate the impact of PBDEs by utilizing high doses for a short duration of time to see the acute effects of this EDC, artificial conditions that are usually not found in actual environmental settings. These investigations have found the association of breast carcinogenesis and progression with PBDE exposure [1, 2, 8]. Although PBDE’s estrogen-like activity has been demonstrated by a handful of previous studies, the biological differences between a high dose (experimental), subacute vs. a low dose (environmentally relevant), chronic PBDE exposure remains largely unknown. Furthermore, previous PBDE studies have predominantly employed the ovariectomy (OVX) mouse models and thus lacked any evaluations during perimenopause period [1, 2, 8]. These circumstances warranted reappraisal of a physiologically relevant animal model to study the perimenopausal mammary gland.

The OVX model, which have been utilized in most mechanistic studies on the external stimuli to reproductive health during the menopausal period, is a surgical procedure that deprives the endogenous sex hormones, such as E2 and P4, by removing the ovaries. However, OVX in mice corresponds to a surgical menopause in women due to the instantaneous decrease of sex hormones, resulting in the disappearance of the perimenopausal state, a critical period in which various organs adjust to the gradual decrease of sex hormones. A more recent model has replaced OVX with a chemically-induced menopause using 4-vinylcyclohexene dioxide (VCD) that promotes gradual follicular apoptosis, which hormonally approximate perimenopausal and later menopausal states with retained ovarian function to secret androgens [9]; so far, these studies mainly focused on metabolic, neuronal, and cardiovascular functions after VCD administration [10, 11]. The experimental protocol and cellular mechanisms by which VCD administration cause chemical menopause have been well established [10, 12–14]. Daily dosing of the chemical for a couple of weeks induced complete follicle loss in the ovary by targeting primordial and primary follicles via direct interaction with cell surface KIT proteins on the oocytes [13]. It has been also demonstrated that VCD does not have secondary pathological toxicity to organs other than the ovary [14, 15]. However, the applicability of the VCD mouse model to study the mammary gland during perimenopausal and menopausal WOS has not been established.

In our study, we hypothesized that the gradual ovarian failure by VCD would provide the mammary gland with perimenopausal and menopausal states. Using such model, we also examined the effects of PBDEs on the mammary gland during and after menopausal transition. PBDEs were exposed to the animals as a mixture of BDE-47, -100, and -153, which were major types of PBDEs found in epidemiological studies and also adopted in the preceding studies [1–3, 8, 16]. BDE-47, -100, and -153 have been shown to act as a weak agonist, a very weak agonist/antagonist, and weak antagonist of estrogen receptor α, respectively [8]. As a result, we found that the VCD mouse model initially provided the mammary gland with moderate regression and less sensitivity to hormonal treatments, which later transformed the glands with fully menopaused characteristics. Single-cell transcriptomic analysis was applied and illustrated that PBDEs could reshape the mammary stroma with environmentally relevant exposures. Taken together, we have demonstrated that the VCD-treated animal is a physiologically relevant model to study mammary gland during this critical WOS, including both perimenopause and menopause states. Furthermore, transcriptome data at the single cell level advanced our understanding of how PBDE exposure could reconstruct the mammary gland in potential association with breast carcinogenesis.

## Methods

### Chemicals and Food Preparation

VCD, 17 β-estradiol, progesterone, dimethyl sulfoxide (DMSO), and sesame oil were purchased from Sigma-Aldrich Corporation, St. Louis, MO. Three PBDE congeners [2,2’,4,4’-tetrabromodiphenyl ether (BDE-47), 2,2’,4,4’,6-pentabromodiphemyl ether (BDE-100), and 2, 2’,4,4′5,5’-hexabromodiphenyl ether (BDE-153)] were purchased from AccuStandard, Inc, New Haven, CT. Fulvestrant was purchased from AstraZeneca, Cambridge, UK.

### Animals

Eight-week-old female C57BL/6 J mice were obtained from the Jackson Laboratory (BarHarbor, ME) and housed in AAALAC-accredited Animal Resources Center, City of Hope. Experimental procedures were approved by the Institutional Animal Care and Use Committee of City of Hope and performed according to the institutional and NIH guidelines for animal care and use. Mice were fed ad libitum a standard mouse chow diet and housed 5 per cage with free access to fresh water and kept on a 12-h light/dark cycle in a conventional clean room. Upon randomization and initiation of PBDE treatment, mice were moved to and housed in polypropylene cages with Sani-Chips beddings 2–3 per cage, and drinking water was filtered twice using reverse osmosis and carbon block system to avoid environmental exposure to potential endocrine-disrupting chemicals [2]. Food consumption was recorded weekly, and total oral exposure to PBDEs were calculated.

### Pilot Evaluation

At 9 w after birth, VCD (130 mg/kg in sesame oil) was intraperitoneally administered 5 sequential days per week for 3 weeks, and treated animals were euthanized at 55, 75, and 130 d post VCD injection based on the previous study [10]. Intact mice received vehicle (sesame oil) injection and were euthanized at the same time points. Two mice per group were included in this pilot experiment. The ovaries, uterus, and mammary gland were collected and routinely processed.

### Mammary Gland Characterization in the VCD-Treated Model

At 9 w after birth, mice were treated with a daily intraperitoneal injection of VCD (160 mg/kg in sesame oil) for 15 d in a row, according to the previous reports [11, 17, 18]. Control mice received vehicle (sesame oil) injection. During the entire experimental period, the animals were fed ad libitum with the control diet (described in the next section) for later comparisons with the PBDE experiments.

*VCD Perimenopause model:* In addition to the five vehicle-treated mice that were used as controls (Intact group), 30 VCD-treated mice were randomized into three groups of VCD, VCD + E2, and VCD + E2 + P4 (n = 10 each) at 7 w post VCD injection. The mice were treated with 17β-estradiol (E2) (1 μg/animal/day) and/or progesterone (P4) (1 mg/animal/day) for seven days as performed in our previous studies [1, 2]. The steroid hormones were dissolved in 100 µL sesame oil and administered intraperitoneally. Non-hormone groups received vehicle injections. Mice were euthanized 24 h after the last injection to collect ovaries, uterus, and mammary glands.

*VCD Menopause model:* 30 VCD-treated mice were randomized into groups of VCD, VCD + E2, and VCD + E2 + P4 (n = 10 each) at 14 w post VCD injection. After another 20 weeks, animals were treated with E2 and P4 for seven days and euthanized as in the Perimenopause model.

### PBDE Exposure Experiments in the VCD Mouse Model

The VCD treatment was performed as described in the *Mammary gland characterization in the VCD-treated model* section. The experimental diet was prepared (Research Diets, Inc, New Brunswick, NJ) to achieve the relevant exposure with the ratio of the three congeners found in human blood (1 mg/kg/day, 0.056 mg/kg/day, and 0.126 mg/kg/day for BDE-47, -100, -153, respectively) [2, 8]. It was designated as the “high subacute” diet. Of note, the previous studies with the same “high subacute” diet resulted in the higher blood concentrations of the PBDE congeners by an order of magnitude compared to the human epidemiological study [1, 2, 8, 16]. In this investigation, we prepared another nutrient-matched diet with 1/20th of the PBDEs concentration (0.05 mg/kg/day, 0.0028 mg/kg/day, and 0.063 mg/kg/day for BDE-47, -100, -153, respectively). This food was designated as the “low chronic” diet. Control diet was made with a special nutrient-matched diet and vehicle (DMSO) (Research Diets, Inc).

*1.Perimenopause High-Subacute PBDE model:* 30 VCD-treated mice were randomized into VCD + PBDE, VCD + E2 + PBDE, VCD + E2 + P4 + PBDE (n = 10 each) at 7 w post VCD injection. The animals were treated with various combinations of the “high subacute” PBDEs treatment diet, 17β-estradiol (1 μg/animal/day), and/or progesterone (1 mg/animal/day) for seven days as performed in our previous studies [1, 2]. The special diet was fed ad libitum to mimic human environmental PBDE exposure via ingestion [19]. Mice were euthanized 24 h after the last injection to collect ovaries, uterus, and mammary glands.

*2.Menopause High-Subacute PBDE model:* 30 VCD-treated mice were randomized into three groups of VCD + PBDE, VCD + E2 + PBDE, and VCD + E2 + P4 + PBDE (n = 10 each) at 14 w post VCD injection. After another 20 weeks, animals were treated E2, P4, and/or “high subacute” PBDEs treatment diet for seven days and euthanized as in the Perimenopause-High PBDE model.

*3.Menopause Low-Chronic PBDE model:* 60 VCD-treated were randomized into three groups of VCD, VCD + PBDE, VCD + E2, VCD + E2 + PBDE, VCD + E2 + P4, and VCD + E2 + P4 + PBDE (n = 10 each) at 14 w post VCD injection. Feeding of “low chronic” PBDE diet was initiated for PBDE groups and continued for the subsequent 20 weeks. Non-PBDE groups were housed in the same special environment (described in the *Animal* section) as the PBDE groups and put on the control diet for these 20 weeks. During the last week on their special diets, animals received designated hormonal treatments for seven days as in the other models. Two mice from the VCD + E2 treatment group were treated with subcutaneous fulvestrant (FUL) injection (5 mg in 100 μL of sterile saline) one day prior to initiation of E2 treatment. These two mice formed an independent VCD + E2 + FUL group for single-cell transcriptomic analysis. Animals were euthanized 24 h after last injection, and the organs were harvested.

### Whole-Mount Mammary Gland Scanning

The glands were fixed with 10% buffered formaldehyde and delipidated with toluene. After rehydration, the glands were stained with 0.025% toluidine blue, and permanent whole-mount staining slides were prepared. Then, the entire glands were captured using Cell^3^iMager Duos (SCREEN Holdings Co., Ltd., Kyoto, Japan) with 20 µm-intervals for the z-axis (Supplementary Fig. 1). Subsequently, the images were segmented with machine learning implementation using Cell^3^iMager Duos and the Model file CC8P06004V00 (SCREEN Holdings Co., Ltd.). The model file has been developed in our previous study [1], in which scanned images with manually labeled of ductal and end buds-like structures were fed into deep learning image analysis to construct an algorithm that automatically segments these structure in a whole-mount mammary gland image. The model file is currently distributed by SCREEN Holdings Co., Ltd. The segmented ductal structures were skeletonized and subjected to branching analysis using the ImageJ software and AnalyzeSkeleton plugin [20, 21], to calculate the total duct length and the number of branching points. The segmented end-buds were also counted using the ImageJ software.

For comparison, the data from our previous study was retrieved [1], in which the ovariectomized animals received E2 treatment (1 μg/animal/day) for seven days 20 weeks after menopause (ovariectomy), paralleling the menopause model in this study.

### Mammary Gland Dissociation And Single-Cell RNA Sequencing

In short, the 4th mammary gland was dissociated with digestion buffer [1.5 mg/mL DNAse I (#10,104,159,001, Millipore Sigma, Burlington, MA), 0.4 mg/mL Collagenase IV (CLS-4, Lot: 47E17528A, Worthington Biochemical Corporation, Lakewood, NJ), 5% FBS, 10 mM HEPES in HBSS]. After dead cells were removed using Dead Cells Removal Microbeads (Miltenyl Biotec, Bergisch Gladbach, Germany), cells with high viability (> 80%) were loaded onto the Chromium Controller (10 × Genomics, Pleasanton, CA), targeting 2,000–5,000 cells per lane. The Chromium v3 single-cell 3′RNA-seq reagent kit (10 × Genomics) generated single-cell RNA-seq libraries. The NovaSeq 6000 system (Illumina, San Diego, CA) sequenced constructed libraries with a depth of 50 k-100 k reads per cell. Raw sequencing data were processed using the 10 × Genomics Cell Ranger pipeline (version 3.1.0) and then aligned to mm10 mouse genome. The datasets can be found in the NCBI GEO database under the accession GSE191219. They have been analyzed by our group for a perspective of fibroblast heterogeneity in the mammary gland, and the results have been published as an independent in-silico analysis [22].

### Data Analysis

The downstream analysis of the scRNAseq analysis was performed using R (ver. 4.1.2) [23] and the Seurat R package (ver. 4.0.5) [24], unless otherwise specified. Raw data were mounted, and low-quality barcodes with < 500 gene count (nFeature_RNA) or > 10% proportion of mitochondrial genes (percent.mt) were filtered. The data from different samples were merged using *merge* function with a default setting. Then, normalization, scaling, variable feature identification, UMAP dimension reduction (*dims* = *30*), and Louvain clustering (*res* = *0.1*) were performed using a standard pipeline according to developer’s vignette. After a sensible clustering was obtained, the DEG analysis and cell cycle scoring were performed using *FindAllMarkers* and *CellCycleScoring* functions, respectively. Annotation of each cluster was determined based on marker gene expression and cell cycle indication.

Pseudo-RNA sequencing data was prepared using *AverageExpression* function. Subsequent clustering and principal component analyses were performed and visualized utilizing *FactoMineR* (ver. 2.4) [25], *factoextra* (ver. 1.0.7) [26], *ape* (ver. 5.5) [27], *ggtree* (ver. 3.2.1) [28] R packages. To unveil the impacts of “low dose” PBDE treatments, gene expression was compared between “VCD” and “VCD + LP”, “VCD + E2” and “VCD + E2 + LP”, “VCD + E2 + P4” and “VCD + E2 + P4 + LP”, individually. Then, commonly regulated genes were extracted from each pair. The same analysis was performed for “high dose” PBDE treatment (+ HP groups). The custom computer scripts and the relevant data are fully available in Zenodo (10.5281/zenodo.7340869) [29]

The upregulated and downregulated genes in either LP or HP samples were further subjected to the Gene Ontology enrichment analysis using The Databese for Annotatrion, Visualization and Integrated Discovery (DAVID) (ver 2021) and the DAVID Knowledgebase (ver v2023q4) [30]. Briefly, the gene lists were uploaded, and the functional annotation charts for GOTERM_BP_DIRECT were retrieved with the default parameter by DAVID.

### Statistical Analysis

All the statistical analyses were performed in R (ver. 4.1.2) and gg*pubr* R package (ver. 0.4.0). Wilcoxon rank-sum test was used to compare the distribution of two conditions of interest. p-value < 0.05 was considered as statistically significant. The number of samples can be found in the figure legends where applicable. The sample size of the study was determined based on the previous study [1, 2].

## Results

### Gradual Mammary Gland Regression in VCD-Induced Menopause Model

A pilot evaluation was performed to preview influences of VCD treatment over time on the mammary gland structure (Supplementary Fig. 2A). Animals were euthanized at 55, 75, and 130 d post VCD injection (130 mg/kg daily, five days per week for three weeks), modeling early perimenopausal, late perimenopausal, and postmenopausal periods, respectively, as previously defined [10]. Whole-mount staining analysis revealed that the mammary glands from 130 d VCD-treated mice had thinner ducts and fewer terminal end bud-like (TEB-L) structures than those in the control mice (Supplementary Fig. 2B). These differences were not evident at the perimenopausal periods (55d and 75d). The results demonstrated that menopause induction by VCD was accompanied with gradual mammary gland regression in mice.

**Fig. 1:**
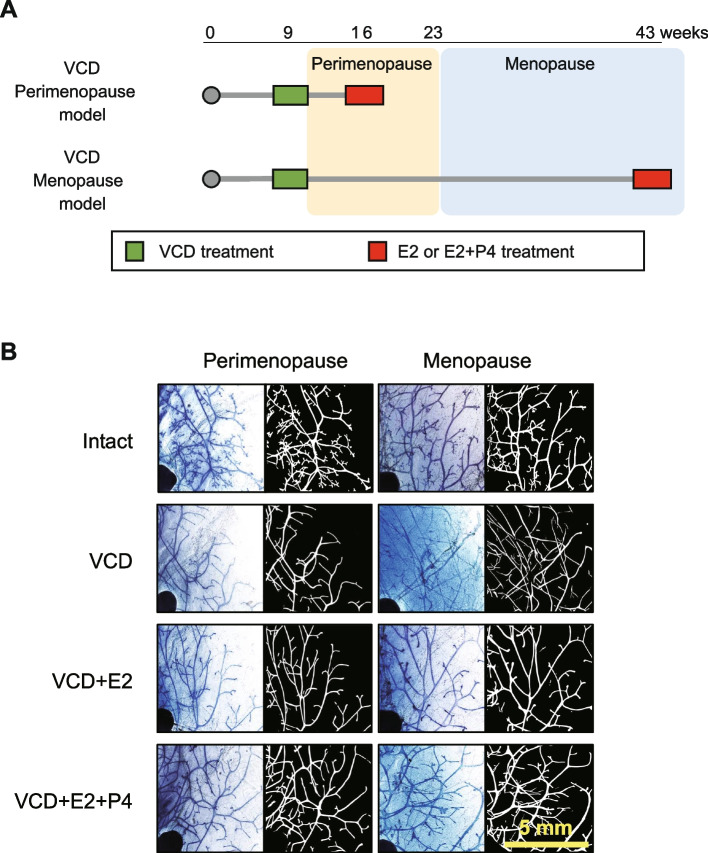
Effects of the VCD treatment on the mouse mammary gland. **A** The overview of the perimenopause and menopause VCD models. VCD treatment was performed at 9 weeks old. The VCD-treated mice were considered to be completely menopaused at 23 weeks old (100 days after the VCD treatment) according to the previous study. **B** Representative images of the mammary gland from the Perimenopause and Menopause VCD models

**Fig. 2:**
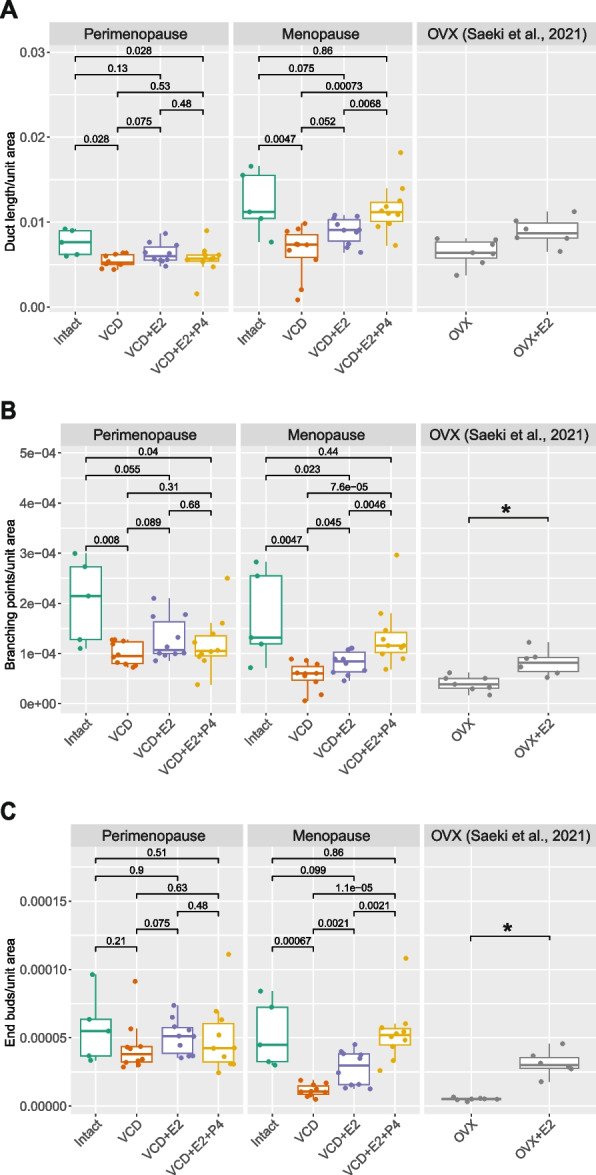
Quantitative analysis of the mouse mammary gland from the Perimenopause and Menopause VCD models. Comparisons of the total ductal length per arbitrary unit area (**A**), the number of the branching points per arbitrary unit area (**B**), and the number of end buds per arbitrary unit area (**C**) between the experimental groups. The box-plot elements were defined as follows: center line, median; box limits, upper and lower quartiles; whiskers, 1.5 × interquartile range; points, outliers. The numbers above brackets indicate *p*-values

Subsequently, the expanded experiment was conducted to characterize the mammary gland during perimenopausal and menopausal period after VCD treatments (Fig. 1A). At this point, we slightly changed the VCD administration to conform to the protocol in which extensive mechanistic and hormonal information is available [17], although the protocol in the pilot study provided detailed subcategorization of perimenopausal periods. Mice were treated with VCD (160 mg/kg for 15 days) and put on the hormonal treatments after the indicated latency period (see Materials and Methods for details). In this model, mice were considered to reach menopause after 100 d post VCD injection based on plateaued blood FSH levels in the previous studies [11, 17]. Responsiveness to external hormonal stimuli was also evaluated premising that hypersensitivity is one hallmark of menopaused mammary glands. In macroscopic evaluation of the whole gland, the VCD group in both Perimenopause and Menopause models showed apparent regression of the gland with the thinner and less terminal end buds (Fig. 1B). With hormonal treatment, the mammary gland regrew with increase in branching and end buds although the effect was less clear in the Perimenopausal model. In the quantitative analysis, the total ductal length and the number of branching points, as well as TEB-L structures, were significantly decreased in the glands of the VCD group in the Menopause model (Fig. 2). Decrease in the total ductal length and branching points were also statistically evident in the Perimenopause group, but to a lesser extent. Treatments of E2 and additional P4 resulted in statistically significant increase in any comparisons in the VCD-treated groups (i.e. VCD vs VCD + E2, VCD + E2 vs VCD + E2 + P4, and VCD vs VCD + E2 + P4) in the Menopause model, except for nearly significant difference in the total duct length between VCD and VCD + E2 groups. Furthermore, after the hormonal treatment, the values were not significantly different compared to those in the intact mice, except for the branching points between Intact and VCD + E2. For comparison, the data from OVX and OVX + E2 groups were retrieved from our previous study [1], which showed that E2 treatment in the Menopause VCD group (considered 20 weeks after VCD-induced menopause) caused a similar regrowth of the gland as in the hypersensitive mammary gland 20 weeks after surgical menopause (OVX). On the other hand, response to the hormonal treatments was not statistically signficant in the Perimenopause gland.

The effects of the VCD treatment on the reproductive organs were examined for model validation. Significant decrease in the ovarian weight was observed in all the groups in the Perimenopause and Menopause models compared to the age-matched vehicle-treated mice (Intact) (Supplementary Fig. 3A). Uterus weights were also significantly decreased in the VCD groups in both model (Supplementary Fig. 3B). Changes in ovarian and uterine weights by VCD treatment were evident in the Perimenopause models, but less so in the Menopause model. Naturally, mice enter the perimenopausal period at about 9 months old [31]. Therefore, older intact mice in the Menopause models (10 months old) had smaller ovaries and uteruses and gained more weight compared to those in the Perimenopause group (4 months old) (Supplementary Figs. 3A-C). Also, the VCD-treated mice in the Menopause model tended to become heavier compared to young and intact animals (Supplementary Fig. 3C), probably due to a less active metabolism from aging and menopause. Notably, the uterine weights increased upon E2 treatment, and then decreased by addition of P4 in the both models, which is the canonical response of the uterus to each hormone. Overall, the experimental procedures in the model development were justified.

Together, at 7 weeks after VCD treatment (the Perimenopause model), the ovary became nearly nonfunctional, however, the mammary gland is still undergoing transition with weaker regression and less sensitivity to the external hormones, probably due to circulating residual hormones in the body. On the other hand, at 20 weeks after previously determined menopause (the Menopause model), the gland is severely regressed and hypersensitive to the hormones as in the OVX model. In brief, the VCD treatment approximated gradual regression of the mammary gland during natural menopausal progression, which enables both perimenopausal and postmenopausal exposures of the mammary gland to external stimuli of interest.

### Peri- vs. Post-Menopausal And Experimental (High-Subacute) vs. Environmental (Low-Chronic) PBDE Exposure

**Fig. 3:**
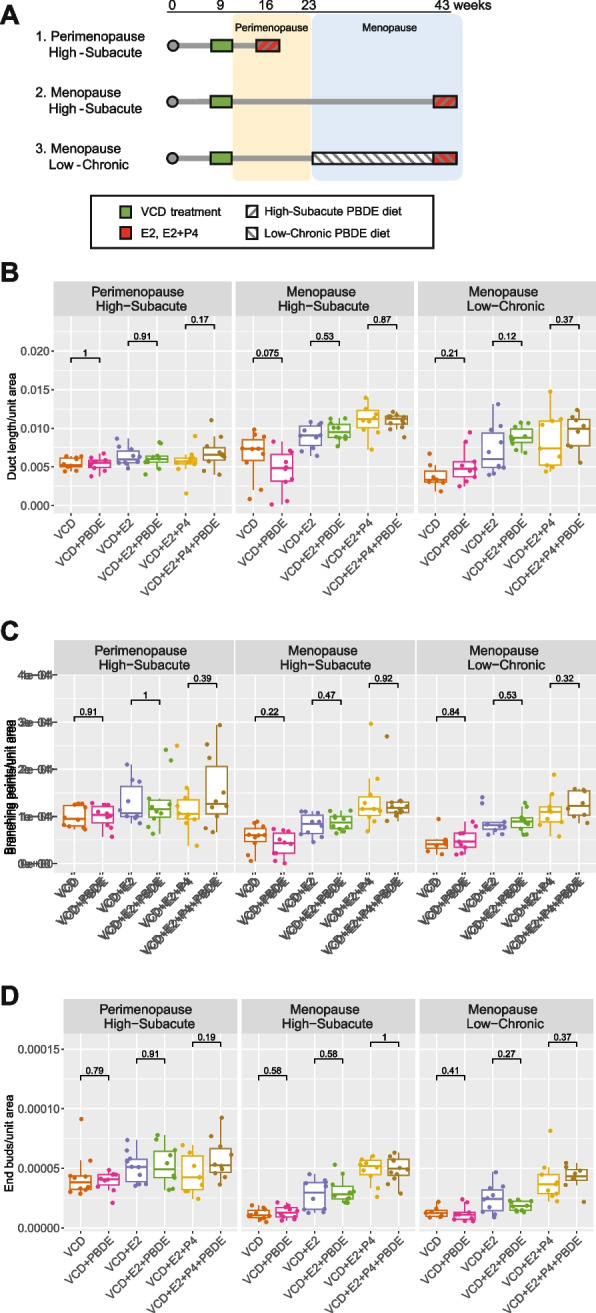
Effects of the PBDEs exposure on the VCD-induced perimenopausal and menopausal mouse mammary gland. **A** The overview of the three different VCD-PBDE experiments. **B-D** Comparisons of the total ductal length per arbitrary unit area (B), the number of the branching points per arbitrary unit area (C), and the number of end buds per arbitrary unit area (D) between the experimental groups. The box-plot elements were defined as follows: center line, median; box limits, upper and lower quartiles; whiskers, 1.5 × interquartile range; points, outliers. The numbers above brackets indicate *p*-values

Leveraging these unique windows of susceptibilities of the mammary gland in the VCD model, we aimed to compare the impact of PBDE exposure on the mammary gland between peri- and post-menopausal exposures (Fig. 3A and Supplementary Fig. 4). Also, the traditional one week-exposure protocol with PBDEs-containing diet (High-Subacute PBDE) resulted in unrealistically high blood concentrations of the PBDEs (Supplementary Fig. 5A). Therefore, in this investigation, we prepared another diet with 1/20th of the PBDE concentration and treated animals for 20 weeks (Low-Chronic PBDE), aiming that the animals will be exposed to environmentally relevant PBDEs concentration. The presumed total amount of PBDE exposure is designed to be the same between the High-Subacute and the Low-Chronic PBDE protocols. Three specifically designed models (Perimenopause High-Subacute PBDE, Menopause High-Subacute PBDE, and Menopause Low-Chronic PBDE) were developed to address the above-described objectives (Supplementary Figs. 5B). Low-Chronic PBDE exposure during perimenopausal period in the VCD model was not possible due to the shorter perimenopausal period in this model as it takes 20 weeks to match the total amount of PBDEs exposure between the Low-Chronic and High-Subacute PBDE models.

Food consumption upon the PBDEs-containing special diets were recorded along with the animal body weight, which enabled estimation of the total amount of oral PBDEs exposure during the model development (Supplementary Fig. 6). Animals ate less food as they got old regardless of the ovarian status (i.e., with or without VCD treatment) (Supplementary Fig. 6A). Also, VCD-treated animals consumed less, especially after presumed menopause, although they gained more weight due to probable impaired metabolism (e.g., decrease estrogen levels) after menopause (Supplementary Figs. 3C and 6B). Special diet with PBDEs did not seem to influence food consuming behavior (no apparent difference between VCD vs VCD + PBDE, VCD + E2 vs VCD + E2 + PBDE, or VCD + E2 + P4 vs VCD + E2 + P4 + PBDE). Although the experiments for the three PBDE models (Perimenopause High-Subacute PBDE, Menopause High-Subacute PBDE, and Menopause Low-Chronic PBDE) were designed to expose animals to the same amount of PBDEs in total, the Menopause High-Subacute PBDE models resulted in slightly smaller estimated exposure to PBDEs (Supplementary Fig. 6C). This was because the Menopause High-Subacute PBDE model was subject to the special diet for one week at 43 weeks old, when mice ate less food, compared to the Perimenopause High-Subacute model (one week at 16 weeks old) and the Menopause Low-Chronic PBDE model (20 weeks at 23–43 weeks old). The ovarian and uterine weight showed no difference by any PBDE exposures, except for the increased ovarian weight in the VCD + PBDE group compared to the VCD group in the Menopause High-Subacute PBDE experiment (Supplementary Fig. 7). Although the previous research described that PBDEs might affect gene expression of ovarian cells [32], the observed difference in the ovarian weight was small, and the significance was not pursued further. The results indicated that PBDEs exposure did not have systemic hormonal influence (E2- or P4-like) in this study.

Macroscopic effects of the treatments on the mammary gland were evaluated through the whole gland staining and subsequent image analysis (Figs. 3B-D and Supplementary Fig. 8). In the three PBDE models, either High-Subacute or Low-Chronic PBDE exposures did not have definitive impact in any combinations macroscopically and in quantitative analyses.

### Post-Menopausal Experimental (High-Subacute) vs. Environmental (Low-Chronic) PBDE Exposure Models: Single-Cell Transcriptomic Analysis

**Fig. 4:**
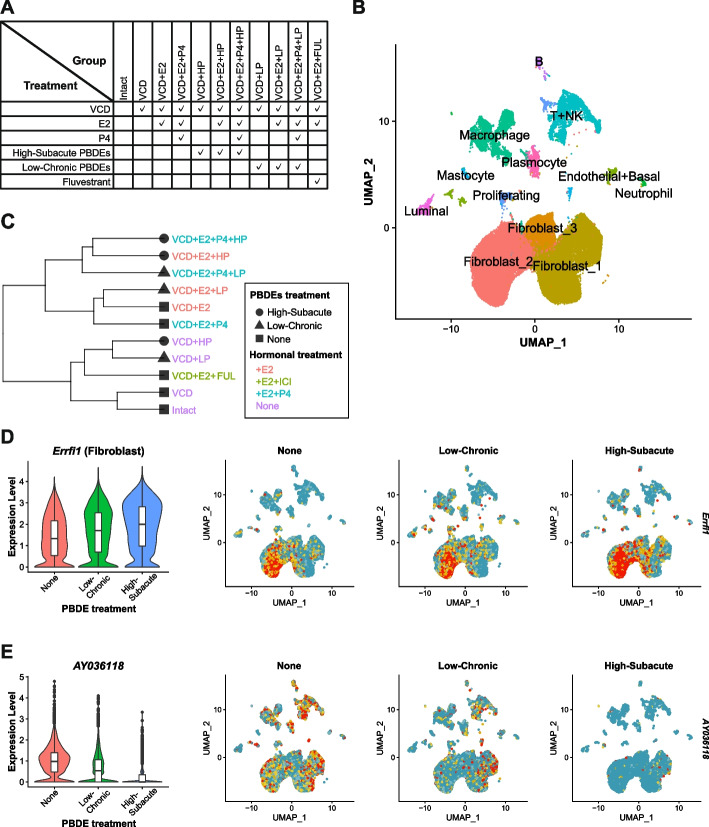
Single-cell RNA sequencing analysis of the whole mammary gland after the VCD treatment, the High-Subacute or the Low-Chronic PBDEs exposure, the hormone replacement treatments, and/or the co-treatment with fluvestrant. **A** The overview of the sample labeling (column) and the experimental interventions (row). **B** The results of dimension reduction and clustering on a UMAP plot with the putative cell type label. **C** The hierarchal clustering of the pseudo RNA seq data. The sample names are accompanied by shapes, which indicate different PBDEs treatments (High-Subacute, Low-Chronic, or None), and color-coded by additional hormonal treatments (+ E2, + E2 + ICI, + E2 + P4, or None). **D-E** The expression of a gene of interest visualized on the violin plot (left) and the UMAP plots (right) stratified and color-coded by the PBDEs exposure. The None group; VCD, VCD + E2, and VCD + E2 + P4. the Low-Chronic group; VCD + LP, VCD + E2 + LP, and VCD + E2 + P4 + LP. The High-Subacute group; VCD + HP, VCD + E2 + HP, and VCD + E2 + P4 + HP. **D***Errfi1*. The violin plot shows expression on *Errfi1* in the fibroblast clusters. **E***AY036118*

Considering that the PBDE exposure did not have a definitive impact on the ductal structures of the mammary gland, we further investigated the cellular and gene expression links of the PBDE exposure to breast carcinogenesis. We performed the scRNAseq analysis of the mammary gland, unbiasedly including all cell types. For scRNAseq analysis, the Menopausal High-Subacute and Low-Chronic models were chosen as the perimenopausal glands were less sensitive to external stimuli. The mammary tissues from two mice in each treatment listed in Fig. 4A were pooled. High-Subacute PBDE- and Low-Chronic PBDE-exposures were abbreviated as HP and LP, respectively. To clarify whether E2’s effects were mediated by ER, an additional VCD + E2 + FUL group was prepared by pre-treating mice with fulvestrant, a selective estrogen receptor degrader, prior to the initiation of E2 treatment.

After initial filtering (nFeature > 500, percent.mt < 10), each sample had comparable quality profiles (Supplementary Fig. 9A). Therefore, all the samples were retained for the subsequent analyses. Initial clustering with an UMAP dimension reduction identified 15 distinct clusters free from severe batch/technical/individual noises (Supplementary Fig. 9B). As no cluster was determined as low-quality based on nFeature_RNA, nCount_RNA, and percent.mt profiles (Supplementary Fig. 9C), the differential gene expression analysis and visualization of characteristic genes on a heatmap and feature plots were performed (Supplementary Figs. 9D and 9E). C (Cluster) 0, 1, and 4 were determined to be fibroblast clusters with *Col1a1* gene expression. C2 had *Lyz2* and *Cd74* expression and was annotated as macrophage. C3 was labeled as T and natural killer (NK) cell cluster based on *Gata3* and *Nkg7* gene expression. C5 showed mixed signatures of multiple cell lineages, such as expression of *Col1a1* and *Gata3*, and was considered a multiplet cluster and removed from further analysis. C6, C9, C10, C11, C12 were annotated as plasma cells, luminal epithelial cells, mast cells, B cells, and neutrophils based on *Jchain*, *Krt18*, *Mcpt4*, *Cd79a*, and *Retnlg* expression, respectively. C7 was composed of two distinct small clusters. One cluster expressed *Pecam1* and the other did *Krt14*, which led to an annotation of endothelial cells + basal cells cluster. C8 was considered as proliferating cells by cell cycle analysis and *Top2a* expression. C13 and C14 were putative nerve sheath cell and striated muscle cell clusters according to *Mpz* and *Acta1* gene expression. However, their cell numbers were too small (< 200), and they were excluded from additional analyses. Eventually, 12 clusters were retained as Fibroblast_1, Fibroblast_2, Macrophage, T + NK, Fibroblast_3, Plasmocyte, Endothelial + Basal, Proliferating, Luminal, Mastocyte, B, and Neutrophil clusters in the order of population size (Fig. 4B). There were no clusters that were found unique in samples or treatments (Supplementary Figs. 10A and 10B).

Pseudo RNA seq conversion and subsequent clustering revealed that E2 treatment had the strongest influences on the whole transcriptome of the mammary gland, with the upper half of the dendrogram exclusively from the estrogen-treated groups. (Fig. 4C). The E2 + FUL sample was clustered together with the non-HRT samples, which confirmed that E2 exerted its effects through ER. In the non-HRT cluster, LP and HP samples formed one subcluster, which indicates common influences by PBDEs exposure. The P4 treatment did not form an isolated cluster. Moreover, in the upper HRT cluster, HP groups were clustered in one node, which also included the LP group with E2 + P4 treatment. These observations indicated that PBDEs treatments had distinct effects on the gland, although their impact was not as strong as estrogen, as anticipated. Also, the High-Subacute PBDEs treatment could have stronger effects compared to the Low-Chronic PBDEs treatment. The results of the principal component analysis further supported the idea (Supplementary Fig. 10C). Two-dimensional visualization by the first two principal components revealed that the non-HRT samples were located in a small area with the VCD + E2 + FUL sample closely neighboring them. In this analysis, the E2 + P4-treated samples had a different distribution compared to the E2 only samples, which indicated progesterone-driven effects on the menopaused gland.

Based on these findings, PBDEs-specific influences were further explored in each cell type. For this analysis, as the major cell type found, the three fibroblast clusters were merged together and renamed as Fibroblast. Briefly, subsets were identified, and differentially expressed genes (DEGs) induced by LP or HP were investigated excluding effects by E2 and P4 (see Materials and Methods). DEGs identified in either LP or HP samples were summarized in Supplementary Table 1 and visualized on violin plots (Supplementary Figs. 11 and 12). As expected, more DEGs were found in the HP samples, especially in Fibroblast, Macrophage, and T + NK clusters. DEGs were hardly identified in epithelial components in both LP and HP samples. In addition, many DEGs in the HP samples were also dysregulated towards the same direction in the LP samples, but to a lower degree. Especially, *Errfi1* in fibroblasts and *AY036118* in macrophages were significantly upregulated and downregulated, respectively, both in the LP and HP (Figs. 4D and E). Except for ribosomal genes, fibroblasts had the greatest number of dysregulated genes that have been associated with carcinogenesis, such as upregulation in *Sepp1*, *Nptn*, and *Ackr3* (Supplementary Table 1). Accordingly, our current analysis revealed that PBDEs could affect non-epithelial components in the mammary gland. Also, the environmentally relevant exposure (Low-Chronic) to PBDEs could exert similar effects as the experimental High-Subacute PBDE exposure.

Furthermore, the Gene Ontology (GO) enrichment analysis was performed to identify specific pathways involved with PBDE exposure. As a result, 68 GO terms were enriched in the upregulated genes, and 36 GO terms were enriched in the downregulated genes (Supplementary Tables 2 and 3). However, these terms were mostly those related to basic cellular processes, and specific pathways known to be dysregulated by the PBDEs, such as female sex hormone signaling and thyroid hormone homeostasis, were not enriched.

Previously, we reported heterogenous fibroblast populations with distinct gene expression signatures in the mouse mammary gland [22]. As fibroblasts had the greatest number of dysregulated genes by PBDEs exposure, advanced subtype analysis was performed. Fibroblasts were subset, and representative marker expression was visualized (Supplementary Figs. 13A and B). Fibroblast_1 cluster was composed of “adipogenic” (F*abp4* +) and “ECM” (*Tnc* +) fibroblasts. Fibroblast_2 cluster corresponded to “interlobular” or “progenitor” (*Dpp4* +) fibroblasts. Fibroblast_3 cluster expressed a marker for “adipo-regulatory” fibroblasts (*F3* +). High-Subacute or Low-Chronic PBDE exposures did not seem to influence distribution of fibroblast subtypes (Supplementary Fig. 13C). *Errfi1* expression was strongest in *Dpp4* + Fibroblast_2 cluster although PBDEs exposure increased its expression in all fibroblast clusters. Therefore, PBDEs did not seem to affect specific fibroblast subtypes.

## Discussion

The OVX mouse model has previously been used to study menopause and the effects of external hormones, such as hormone replacement therapy and environmental pollutant exposures. However, the implementation of OVX on mice is not an accurate representation of natural human menopause due to its surgical nature and instantaneous depletion of sex hormones. More recently, VCD has been used to study premature ovarian insufficiency because it gradually destroys the ovarian follicles, promoting ovarian failure in mice [9]. VCD treatment retains androgen production by the ovaries. Thus, the introduction of VCD simulates natural human menopause more accurately, allowing for the structural and cellular reconstruction of the organs to be examined over time as ovarian functions gradually cease. The accepted consensus is that if the ovarian follicles are destroyed, the mammary gland would regress due to the decrease of circulating levels of sex hormones, such as E2 and P4. Previously, it was found that the circulating female hormone levels significantly dropped at day 127 from the onset of the same VCD treatment [17] (control vs VCD-treated, P4: 4.54 ± 0.07 ng/ml vs 3.39 ± 0.06 ng/ml, P4 2.21 ± 0.05 pg/ml vs undetectable). Our current study, including both macroscopic and single-cell level analyses, is the first documented study of the VCD model focusing on the mammary gland. The results demonstrated that VCD treatment led to gradual mammary gland regression with reduced mammary gland ductal lengths and fewer branching points. Additionally, E2 and P4 could induce significant regrowth of the ductal structures in the VCD-induced menopausal mammary glands, as expected, to the level statistically insignificant to those in the intact mice. This result robustly supported our hypothesis that the mammary gland regression occurs due to decrease in circulating hormone levels after ovarian failure, rather than direct effects of VCD on the gland. In contrast, perimenopausal mammary glands were less responsive to external hormone exposure, probably due to higher levels of sex hormones remaining in the glands.

Although PBDEs have been linked to breast carcinogenesis risk, the exact long-term effects are still being defined experimentally. By leveraging the effects of VCD-induced ovarian follicle depletion, we could determine the subtle effects of PBDEs exposure in the mammary gland during the physiologically-relevant perimenopause and menopause periods. Regarding effects on the menopausal mammary gland, to our best knowledge, the previous studies in the rodents have focused on PBDE exposures at the experimental level by utilizing a high dose of PBDEs for a short duration of time [1, 2, 8]. Such studies have not been expanded into long-term, low PBDE dose exposure to replicate the persistent environmental exposure to this EDC. Here, we compared the differences in a short-term, high PBDE dose (High-Subacute) versus a long-term, low PBDE dose (Low-Chronic) to reveal the implications of “experimental” and “environmental” PBDEs exposure on mammary glands, through a VCD-induced menopausal transition model.

Across the three PBDE treatment models, the ovary and uterus weights were not severely affected by different PBDE concentrations and exposure durations. Also, the effects of PBDEs on the macroscopic structure of mammary glands were not detectable regardless of the varying treatment protocols or cotreatment with the sex hormones. Although PBDE congeners were identified as agonists or antagonists of ER and PR in vitro [8], the current observations indicated that in vivo effects of PBDEs on the mammary epithelium (associated with the ductal structure) may not be as drastic as previously considered, regardless of exposure doses and durations (experimental and environmental exposures).

Recent technical advances have enabled a comprehensive gene expression assessment in the tissue at an unprecedented level, which would reveal the otherwise hidden subtle and complex effects of environmental exposure [33]. In this study, the scRNAseq analysis successfully revealed dysregulation of explicit genes in specific cell populations upon the PBDEs exposure. The results also indicated that Low-Chronic PBDEs exposure exerts similar effects as High-Subacute PBDEs exposure, but to a lesser extent, signifying that environmental PBDE exposure could be hazardous.

Fibroblasts are major players in mammary gland organization and have also been repeatedly associated with breast carcinogenesis [22, 34]. Upregulation of *Errfi1* was observed in fibroblasts after both low and high PBDEs treatments (Fig. 5D). Previous studies showed that *Errfi1* is selectively downregulated in cancer tissues, including breast, thyroid, and lung cancers, and its expression is associated with better prognosis, suggesting *Errfi1* as a tumor suppressor gene [35, 36]. Interestingly, specific expression of *Errfi1* in fibroblasts is associated with cellular senescence [37], and senescent fibroblasts have been related to promotion of neoplastic epithelial growth [38, 39]. Therefore, specific *Errfi1* induction in fibroblasts might be in favor of breast carcinogenesis. Upregulated genes in fibroblasts by High dose PBDE exposure included *Sepp1, Nptn,* and *Ackr3* (Supplementary Table 1), which have also been associated with cancer development and progression [40] [41, 42] [43, 44] [45]. Significance of each change need to be determined in the future.

Our previous scRNAseq study revealed that estrogen together with High-Subacute PBDEs exposure might increase M2-polarized macrophages [2], which facilitated our analytical focus on this cell type. In this study, we identified the consistent downregulation of *AY036118* in macrophages by low and high dose PBDEs exposure. *AY036118* is also known as *Erf1*, an ETS transcription factor, that suppresses ETS-associated tumorigenesis [46]. The ETS family of transcription factors is known to drive oncogenic signaling from kinases, and ETS factor expression is aberrantly upregulated in solid tumors through chromosomal translocation and amplification [47]. Therefore, *AY036118* downregulation in cells might lead to abnormal cell growth. However, *AY036118* was rather ubiquitously dysregulated in multiple cell types, not specifically in macrophages. Additionally, a recent report described that *AY036118* and *GM42418*, which is another ubiquitously downregulated gene concurrently with *AY036118* in our data, overlap the rRNA element Rn45s and represent rRNA contamination [48]. Therefore, our current analysis points out a need for future investigation of the biological meaning of changes in *AY036118* and other ribosome-associated genes, such as *GM42418*, *Uba52*, and *Rps*s in mammary glands.

Our study did have several limitations. First, the circulating hormone levels have not been measured after the VCD treatment in this study although the extensive characterization of the same model had been previously performed [17]. Second, the scRNAseq data was only conducted on menopausal mammary glands. Dynamic gland regression during perimenopause could render the gland unique vulnerability at the molecular level to the EDCs. Third, the scRNAseq data had a limited number of epithelial cells in this study, which might have masked changes in this compartment. This points out the isolation of cells during the single-cell process requires additional advancement. Fourth, the experimental design could be elaborated more so that all the groups have the same exposure in total, taking food consuming tendency with age into account. Finally, our study is at the gene expression level. Future studies may focus on whether PBDE-mediated *Errfi1* expression and potential senescence in fibroblasts could promote breast carcinogenesis.

In conclusion, we successfully confirmed that the VCD treatment can provide a physiologically relevant model to study unique WOSs of the mammary gland, namely perimenopausal and menopausal periods. Our study has shown that mammary glands at the postmenopausal stage are more sensitive to estrogen/progesterone and their mimics than the glands at the perimenopausal period. Utilizing this model, we found that low dose PBDE exposure also exerts similar effects as high dose PBDE, but to a lesser extent. This would indicate that environmental exposure can affect the human breast tissue. Our analysis at the single-cell level revealed novel molecular effects of PBDEs on non-ER pathways and non-epithelial compartments. Future studies should follow to reveal whether and how these mechanisms can lead to breast carcinogenesis.

### Supplementary Information

Supplementary Material 1.

## Data Availability

The authors declare that all data supporting the findings of this study are available within the article and data repositories described below or from the corresponding author upon reasonable request. The scRNAseq data obtained in this study were deposited in the Gene Expression Omnibus along with their associated meta data (https://www.ncbi.nlm.nih.gov/geo/, accession number GSE191219). The merged data are explorable on the web browser and can be downloaded as Seurat R objects at https://mouse-mammary-pbde.cells.ucsc.edu/ (UCSC cell browser). The relevant data and custom codes in this study were deposited and available in Zenodo (10.5281/zenodo.7340869).
