# Supplementary material for: Perimenopausal and Menopausal Mammary Glands In A 4-Vinylcyclohexene Diepoxide Mouse Model
Source: J Mammary Gland Biol Neoplasia. 2024 Jul 17;29(1):15. doi: 10.1007/s10911-024-09569-x (PMC11254995; doi:10.1007/s10911-024-09569-x)
Supplement: Supplementary file 1 — Supplementary Material 1. [file 10911_2024_9569_MOESM1_ESM.pdf]

Supplementary Information

Journal of Mammary Gland Biology and Neoplasia

Perimenopausal and menopausal mammary glands in a 4-vinylcyclohexene diepoxide mouse model

Kohei Saeki[1,2], Desiree Ha[1], Gregory Chang[1], Hitomi Mori[1], Ryohei Yoshitake[1], Xiwei Wu[3], Jinhui Wang[3], Yuan-Zhong Wang[1], Xiaoqiang Wang[1], Tony Tzeng[1], Hyun Jeong Shim[1], Susan L Neuhausen[4], Shiuan Chen[1]

1. Department of Cancer Biology and Molecular Medicine, Beckman Research Institute of City of Hope, Duarte, CA, USA

2. Faculty of Veterinary Medicine, Okayama University of Science, Ehime, JAPAN

3. Integrative Genomics Core, Beckman Research Institute of City of Hope, Duarte, CA, USA,

4. Department of Population Sciences, Beckman Research Institute of City of Hope, Duarte, CA, USA

Correspondence: Shiuan Chen, Ph.D.

Department of Cancer Biology and Molecular Medicine

Beckman Research Institute of the City of Hope

1500 East Duarte Road, Duarte, CA, 91010

Tel: (626) 218-3454, Fax: (626) 301-8972

E-mail: schen@coh.org

Supplementary Tables

Supplementary Table 1 Differentially expressed genes by the two PBDE conditions.

Cluster	Up/ Down	Low PBDE	High PBDE
Fibroblast	Up	*Dnajb1, Errfi1, Hspa1b*	*Eef2, Sepp1, Gstm1, Nptn, Serping1, Eef1a1, Gm12840, Ackr3, Plpp3, Galnt15 ,Eif3f, Pcolce2, Npr1, Scara3, Htra3, Creb5, Ddr2, Chp1, Notch2, Car8, Ypel3, Zbtb16, Fn1, Fosl2, Errfi1, Hes1, Igfbp4, Cxcl13, Tiparp*
	Down	-	*Uba52, Gm42418, AY036118, Rps29, Lars2, Rps23, Tmsb10, Rps28, Rps27, Gm10073, Rps18, Rps17, Gm10076, Bloc1s1, Rpl37a, mt-Atp8, Bst2, Icam1, Txn1, Casp4, Slfn5, Sparcl1, Gem, Ifrd1, Mgp, Cxcl1, Idi1, Hmgcs1*
Endothelial+Basal	Up	-	-
	Down	-	*AY036118*
Neutrophil	Up	-	-
	Down	-	-
Macrophage	Up	*Mt2*	*mt-Nd4, Tsc22d3, mt-Cytb, Tubb5, Glul, Tuba1c*
	Down	*AY036118*	*Uba52, Gm42418, AY036118, Rpl35, Rps29, Rpl38, Rps27, Rps18, Gm10073, Rps6, Rps23, Txn1, Tmsb10, Rpl13a, S100a11, Tln1*
T+NK	Up	-	*Eef2, Gnb1, Gnai2, Actg1, Cd3e*
	Down	-	*Uba52, Gm42418, AY036118, Rps29, Rps27, Rpl35, Rps23, Rpl38, Rps21, Rps28, Rps14, Rps18, Gm10073, Rps6, Rpl26, mt-Atp8, Gsn, Col3a1, Rps17, Rplp2, Rpl37a, Rps25, Prkca, Lars2, Atp5e, Rpl31*
Mastocyte	Up	-	-
	Down	-	-
Proliferating	Up	-	-
	Down	-	-

B	Up	-	-
	Down	-	-
Luminal	Up	-	-
	Down	-	-
Plasmocyte	Up	-	-
	Down	-	*AY036118, Gm42418*

Supplementary Table 2 Gene ontology analysis of the upregulated genes by the PBDE exposure

**Term**	**Count**	**Adjusted p-value**
GO:0016310~phosphorylation	17	5.51E-03
GO:0042325~regulation of phosphorylation	14	5.58E-03
GO:0001932~regulation of protein phosphorylation	13	6.35E-03
GO:0019220~regulation of phosphate metabolic process	14	6.35E-03
GO:0051174~regulation of phosphorus metabolic process	14	6.35E-03
GO:0006468~protein phosphorylation	14	1.07E-02
GO:0008283~cell proliferation	15	1.07E-02
GO:0051247~positive regulation of protein metabolic process	13	1.07E-02
GO:0051246~regulation of protein metabolic process	17	1.07E-02
GO:0070372~regulation of ERK1 and ERK2 cascade	7	1.07E-02
GO:0019538~protein metabolic process	24	1.07E-02
GO:0048584~positive regulation of response to stimulus	16	1.07E-02
GO:1902533~positive regulation of intracellular signal transduction	11	1.25E-02
GO:0007166~cell surface receptor signaling pathway	16	1.25E-02
GO:0032268~regulation of cellular protein metabolic process	16	1.25E-02
GO:0007167~enzyme linked receptor protein signaling pathway	10	1.25E-02
GO:0031399~regulation of protein modification process	13	1.37E-02
GO:0006796~phosphate-containing compound metabolic process	17	1.43E-02
GO:0010604~positive regulation of macromolecule metabolic process	18	1.46E-02
GO:0032270~positive regulation of cellular protein metabolic process	12	1.47E-02
GO:0042221~response to chemical	23	1.57E-02
GO:0006793~phosphorus metabolic process	17	1.57E-02
GO:0001944~vasculature development	9	1.57E-02
GO:0072358~cardiovascular system development	9	1.57E-02
GO:0010033~response to organic substance	19	1.57E-02
GO:0042127~regulation of cell proliferation	13	1.57E-02
GO:0048583~regulation of response to stimulus	20	1.57E-02
GO:0012501~programmed cell death	14	1.57E-02
GO:0009628~response to abiotic stimulus	11	1.57E-02

GO:0008219~cell death	14	1.57E-02
GO:0009719~response to endogenous stimulus	13	1.57E-02
GO:1902531~regulation of intracellular signal transduction	13	1.57E-02
GO:0044710~single-organism metabolic process	19	1.68E-02
GO:0044267~cellular protein metabolic process	21	1.69E-02
GO:0032964~collagen biosynthetic process	4	1.69E-02
GO:0010562~positive regulation of phosphorus metabolic process	10	1.69E-02
GO:0045937~positive regulation of phosphate metabolic process	10	1.69E-02
GO:0009893~positive regulation of metabolic process	18	2.30E-02
GO:0031401~positive regulation of protein modification process	10	2.35E-02
GO:0048518~positive regulation of biological process	25	2.35E-02
GO:0033631~cell-cell adhesion mediated by integrin	3	2.35E-02
GO:0006915~apoptotic process	13	2.42E-02
GO:0006464~cellular protein modification process	17	2.60E-02
GO:0070371~ERK1 and ERK2 cascade	6	2.60E-02
GO:0001934~positive regulation of protein phosphorylation	9	2.60E-02
GO:0035988~chondrocyte proliferation	3	2.60E-02
GO:0044763~single-organism cellular process	34	2.60E-02
GO:0036211~protein modification process	17	2.60E-02
GO:0006950~response to stress	19	2.60E-02
GO:0043408~regulation of MAPK cascade	8	2.60E-02
GO:0019932~second-messenger-mediated signaling	6	2.60E-02
GO:0022409~positive regulation of cell-cell adhesion	6	2.61E-02
GO:0045785~positive regulation of cell adhesion	7	2.68E-02
GO:0048522~positive regulation of cellular process	23	2.87E-02
GO:0070887~cellular response to chemical stimulus	17	2.87E-02
GO:0001568~blood vessel development	8	3.15E-02
GO:0042327~positive regulation of phosphorylation	9	3.19E-02
GO:0000165~MAPK cascade	8	3.31E-02
GO:0023014~signal transduction by protein phosphorylation	8	3.31E-02
GO:0050896~response to stimulus	32	3.95E-02
GO:0043412~macromolecule modification	17	3.95E-02
GO:0044237~cellular metabolic process	32	3.98E-02
GO:0030335~positive regulation of cell migration	7	4.11E-02

GO:0070374~positive regulation of ERK1 and ERK2 cascade	5	4.50E-02
GO:2000147~positive regulation of cell motility	7	4.96E-02
GO:0048519~negative regulation of biological process	22	4.96E-02
GO:0080090~regulation of primary metabolic process	23	4.96E-02
GO:0035556~intracellular signal transduction	14	4.96E-02
GO:0009967~positive regulation of signal transduction	11	5.51E-03
GO:0008284~positive regulation of cell proliferation	9	5.58E-03
GO:0031325~positive regulation of cellular metabolic process	16	6.35E-03
GO:0032963~collagen metabolic process	4	6.35E-03
GO:0044259~multicellular organismal macromolecule metabolic process	4	6.35E-03
GO:0051272~positive regulation of cellular component movement	7	1.07E-02

Supplementary Table 3 Gene ontology analysis of the downregulated genes by the PBDE exposure

Term	Count	Adjusted p-value
GO:0002181~cytoplasmic translation	17	8.83E-20
GO:0006412~translation	20	4.00E-13
GO:0043043~peptide biosynthetic process	20	4.57E-13
GO:0043604~amide biosynthetic process	20	2.52E-12
GO:0006518~peptide metabolic process	20	8.26E-12
GO:1901566~organonitrogen compound biosynthetic process	22	3.17E-11
GO:0043603~cellular amide metabolic process	20	2.16E-10
GO:0042274~ribosomal small subunit biogenesis	9	1.43E-08
GO:0000028~ribosomal small subunit assembly	6	9.90E-08
GO:1901564~organonitrogen compound metabolic process	22	9.90E-08
GO:0042254~ribosome biogenesis	11	1.48E-07
GO:0022613~ribonucleoprotein complex biogenesis	12	4.82E-07
GO:0006364~rRNA processing	9	1.16E-06
GO:0016072~rRNA metabolic process	9	3.53E-06
GO:0042255~ribosome assembly	6	2.61E-05
GO:0022618~ribonucleoprotein complex assembly	8	6.19E-05
GO:0034470~ncRNA processing	9	6.19E-05
GO:0034660~ncRNA metabolic process	10	6.19E-05
GO:0071826~ribonucleoprotein complex subunit organization	8	1.03E-04
GO:0044267~cellular protein metabolic process	25	1.37E-04
GO:0044271~cellular nitrogen compound biosynthetic process	25	3.30E-04
GO:0019538~protein metabolic process	26	4.74E-04
GO:0044085~cellular component biogenesis	20	5.36E-04
GO:0044249~cellular biosynthetic process	27	7.88E-04
GO:0034622~cellular macromolecular complex assembly	12	8.13E-04
GO:0043933~macromolecular complex subunit organization	15	8.61E-04
GO:0065003~macromolecular complex assembly	14	0.001153
GO:0009058~biosynthetic process	27	0.001205
GO:1901576~organic substance biosynthetic process	26	0.003142

GO:0010467~gene expression	25	0.004647
GO:0034645~cellular macromolecule biosynthetic process	22	0.016518
GO:0006396~RNA processing	9	0.016518
GO:0009059~macromolecule biosynthetic process	22	0.019115
GO:0022607~cellular component assembly	16	0.026659
GO:0034641~cellular nitrogen compound metabolic process	25	0.036549
GO:0071840~cellular component organization or biogenesis	25	0.044749

Supplementary Figures

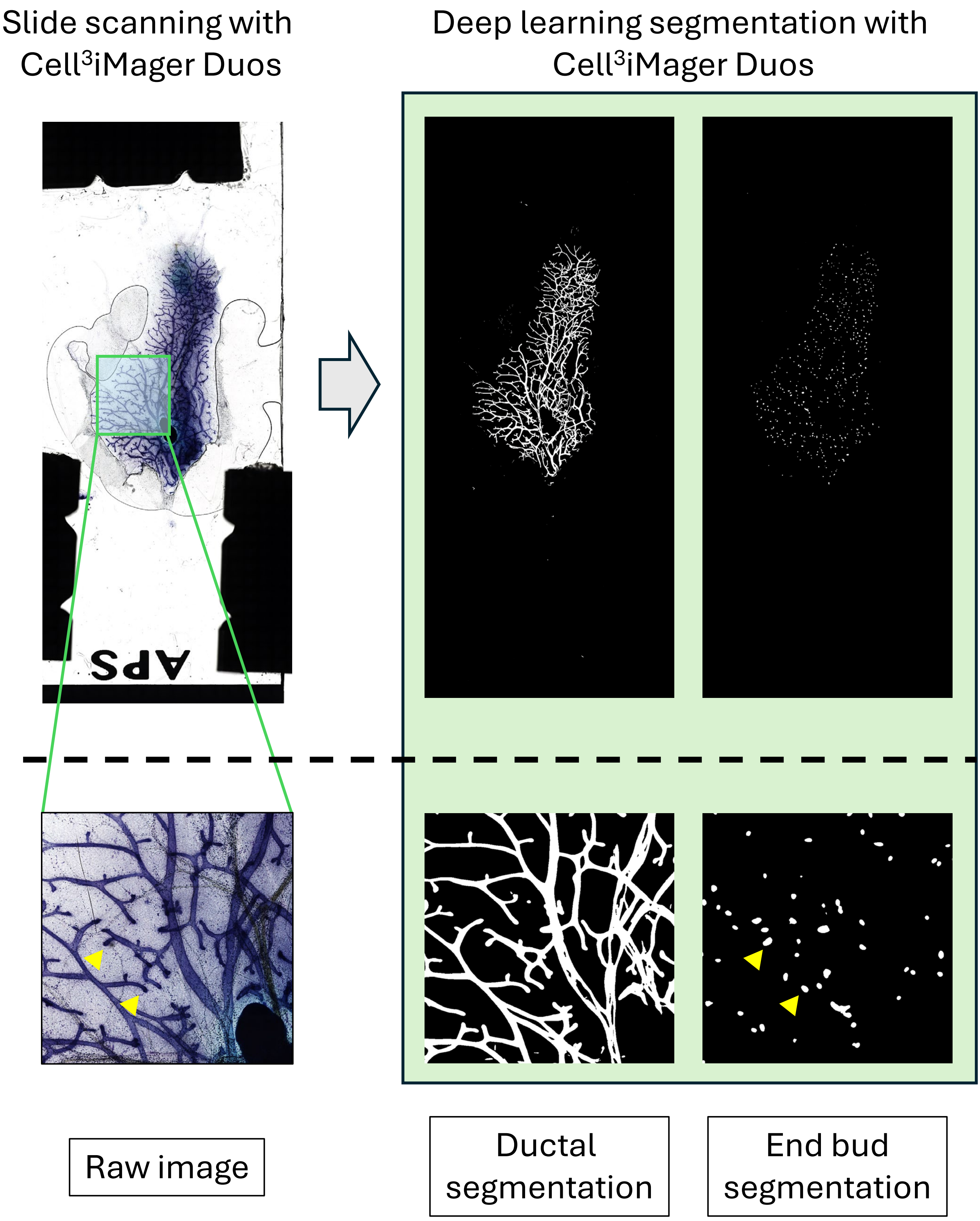
Slide scanning with
Cell³iMager Duos
Deep learning segmentation with
Cell³iMager Duos
APS
Raw image
Ductal
segmentation
End bud
segmentation

Supplementary Figure 1 Deep learning image analysis of the whole-mount mammary gland images. A whole-mount mammary gland slide was scanned with Cell^{3}iMager Duos, and then subject to deep learning analysis to segment ductal and end bud-like structures using the same instrument and the previously developed model algorithm. The segmented images were further analyzed with the ImageJ software. Top panels, raw and segmented image of a mammary gland whole mount. Bottom panels, raw and segmented image of the arbitrary magnified area. Yellow arrow heads, selected end bud-like structures for a comparison between the raw and segmented images.

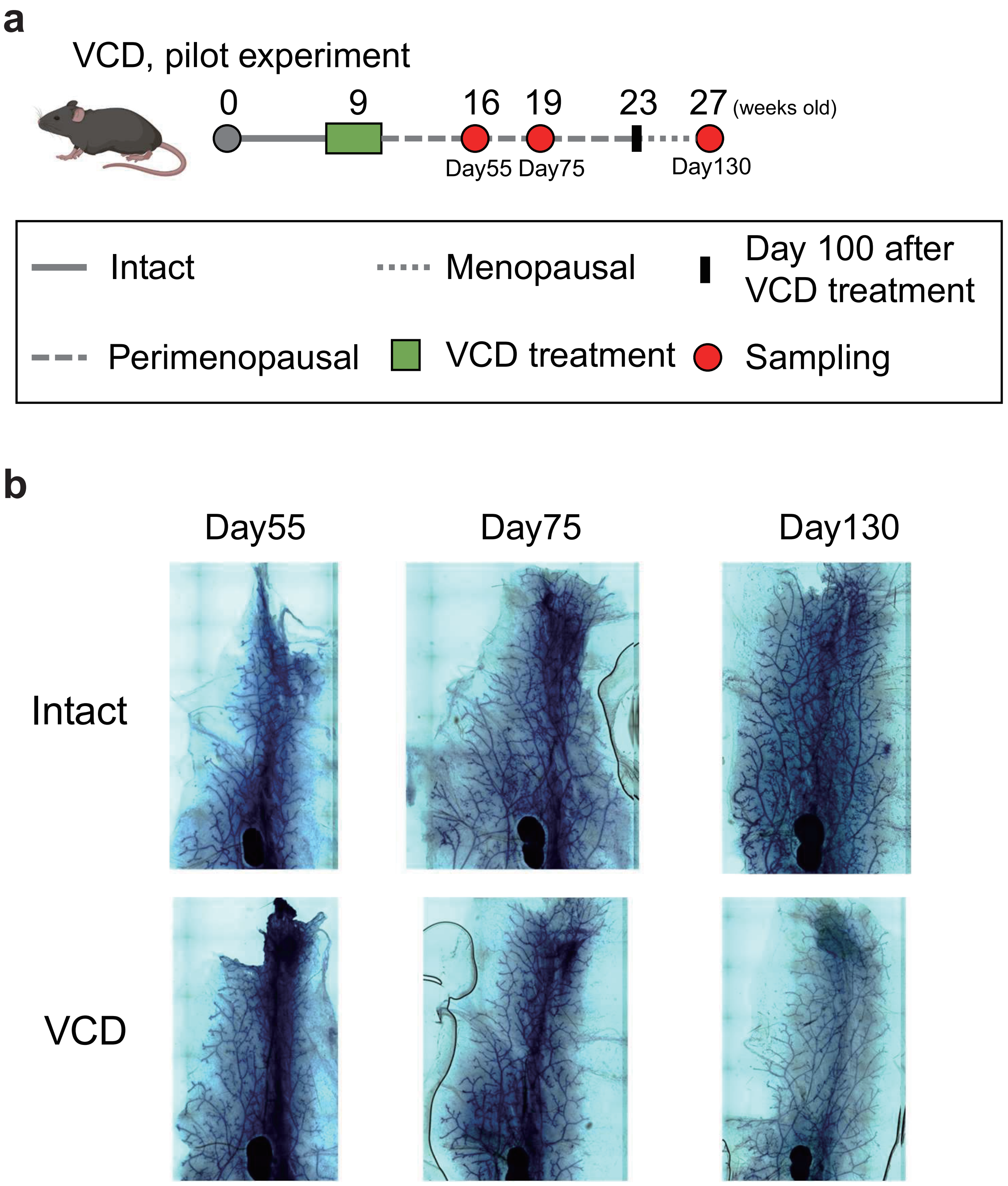

a
VCD, pilot experiment
0
9
16
19
23
27 (weeks old)
Day55 Day75
Day130
Intact
Menopausal
Day 100 after VCD treatment
Perimenopausal
VCD treatment
Sampling
b
Day55
Day75
Day130
Intact
VCD

Supplementary Figure 2 The pilot VCD evaluation. (A) The overview of the experiment. **(B)** The representative whole gland staining images from the Intact (vehicle-treated) and the VCD group at each sampling point.

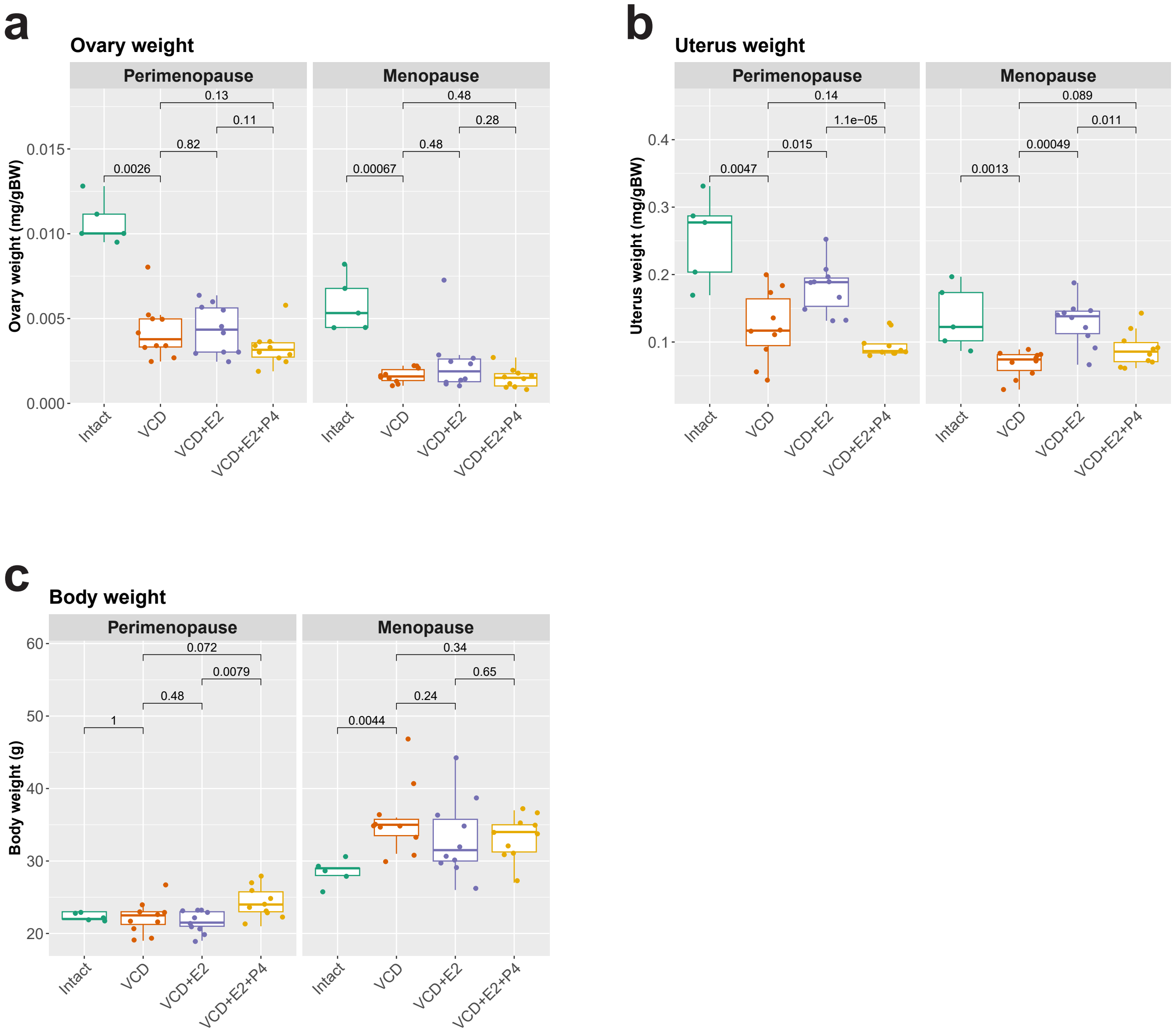

a
Ovary weight
Perimenopause
Menopause
Ovary weight (mg/gBW)
0.000
0.005
0.010
0.015
0.0026
0.82
0.13
0.11
0.00067
0.48
0.48
0.28
Intact
VCD
VCD+E2
VCD+E2+P4
b
Uterus weight
Perimenopause
Menopause
Uterus weight (mg/gBW)
0.1
0.2
0.3
0.4
0.0047
0.015
0.14
1.1e−05
0.0013
0.00049
0.089
0.011
c
Body weight
Perimenopause
Menopause
Body weight (g)
20
30
40
50
60
1
0.48
0.072
0.0079
0.0044
0.24
0.34
0.65

Supplementary Figure 3 Effects of the VCD and the hormone replacement treatments on the ovarian (A), the uterine (B), and body weight (C). The box-plot elements were defined as follows: center line, median; box limits, upper and lower quartiles; whiskers, 1.5x interquartile range; points, outliers. The numbers above brackets indicate p-values.

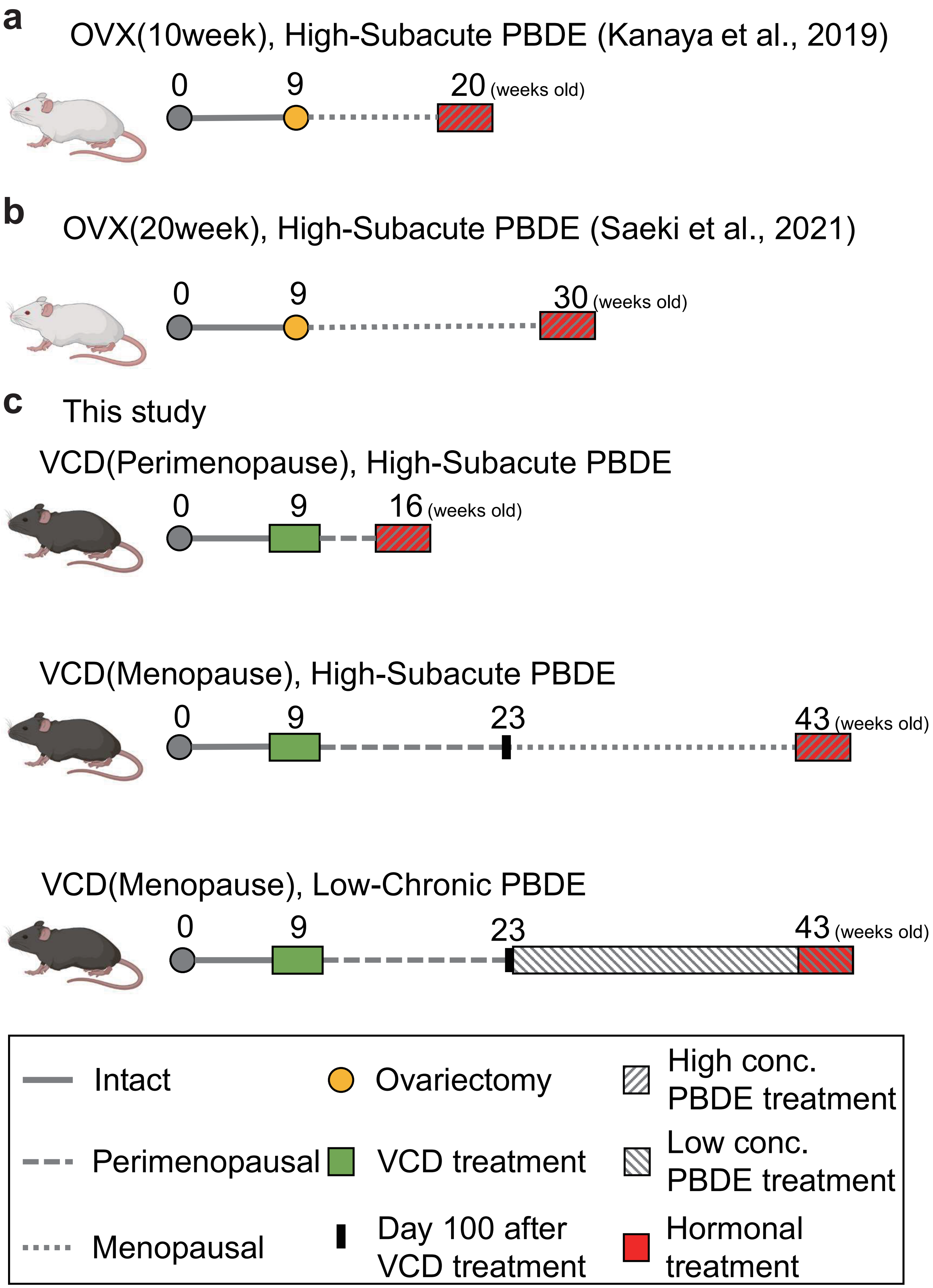
a
OVX(10week), High-Subacute PBDE (Kanaya et al., 2019)
0
9
20 (weeks old)
b
OVX(20week), High-Subacute PBDE (Saeki et al., 2021)
0
9
30 (weeks old)
c
This study
VCD(Perimenopause), High-Subacute PBDE
0
9
16 (weeks old)
VCD(Menopause), High-Subacute PBDE
0
9
23
43 (weeks old)
VCD(Menopause), Low-Chronic PBDE
0
9
23
43 (weeks old)
Intact
Ovariectomy
High conc. PBDE treatment
Perimenopausal
VCD treatment
Low conc. PBDE treatment
Menopausal
Day 100 after VCD treatment
Hormonal treatment

Supplementary Figure 4 The comparisons of the experimental protocols between the preceding studies and the current study. (A) Kanaya et al, 2019 [1]. **(B)** Saeki et al, 2021 [2]. **(C)** The current study.

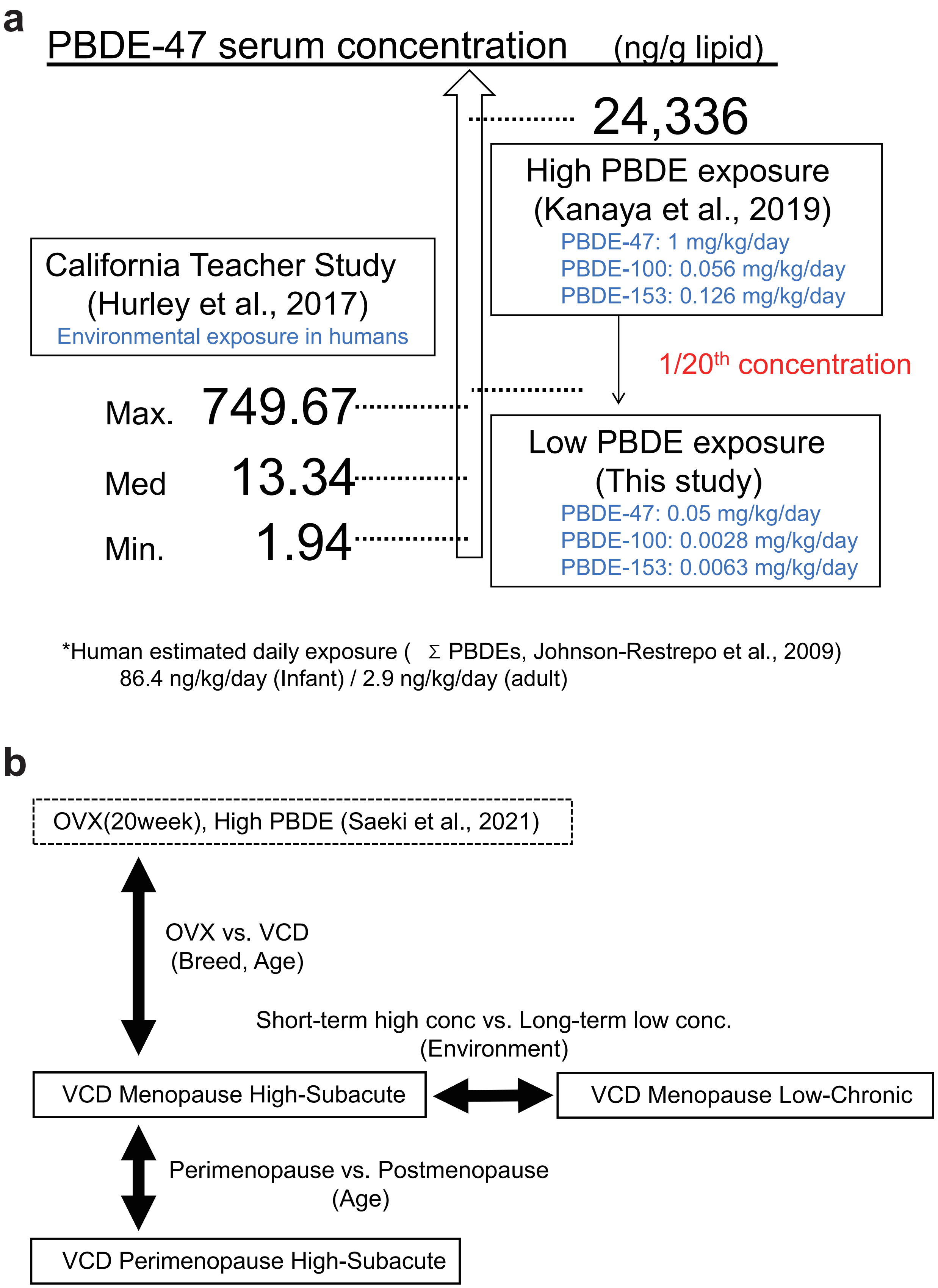
a
PBDE-47 serum concentration (ng/g lipid)
24,336
High PBDE exposure
(Kanaya et al., 2019)
PBDE-47: 1 mg/kg/day
PBDE-100: 0.056 mg/kg/day
PBDE-153: 0.126 mg/kg/day
California Teacher Study
(Hurley et al., 2017)
Environmental exposure in humans
1/20th concentration
Max. 749.67
Med 13.34
Min. 1.94
Low PBDE exposure
(This study)
PBDE-47: 0.05 mg/kg/day
PBDE-100: 0.0028 mg/kg/day
PBDE-153: 0.0063 mg/kg/day
*Human estimated daily exposure (Σ PBDEs, Johnson-Restrepo et al., 2009)
86.4 ng/kg/day (Infant) / 2.9 ng/kg/day (adult)
b
OVX(20week), High PBDE (Saeki et al., 2021)
OVX vs. VCD
(Breed, Age)
Short-term high conc vs. Long-term low conc.
(Environment)
VCD Menopause High-Subacute
VCD Menopause Low-Chronic
Perimenopause vs. Postmenopause
(Age)
VCD Perimenopause High-Subacute

Supplementary Figure 5 Factors for the experimental design of the current study. (A) The PBDEs exposure protocol. The preceding study by Kanaya et al. [1] adopted the High-Subacute PBDEs exposure protocols which resulted in more than 30 times higher serum concentration of the PBDE-47 compared to a human epidemiological study (the California Teacher Study) [3]. In the current study, the Low-Chronic PBDEs exposure protocol was prepared to mimic a biologically-relevant exposure. **(B)** Potential comparisons between the protocols in the preceding study and the current study. The words in bold represent factors that can be compared between the protocols. The words in parenthesis represent potential confounding factors.

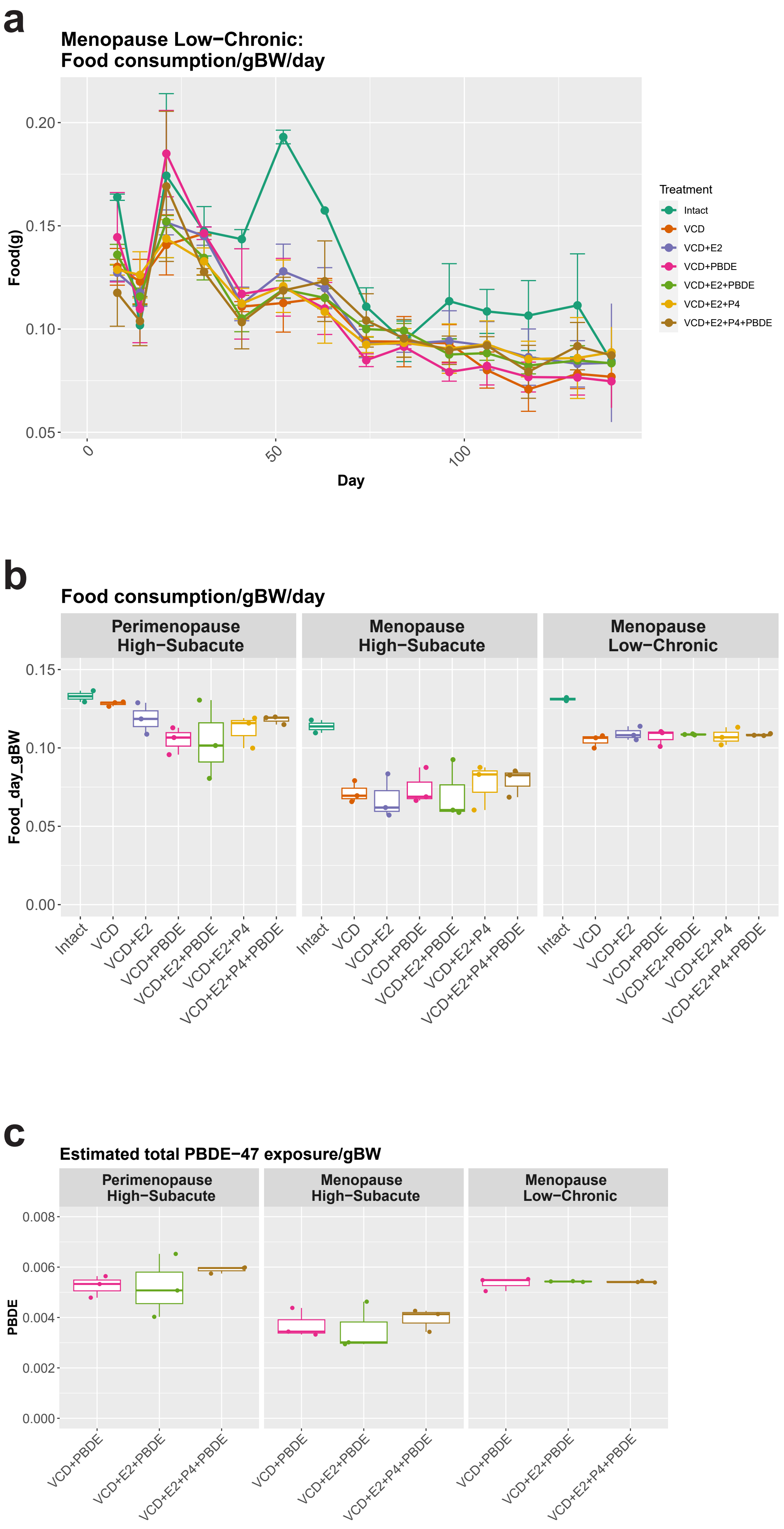

a
Menopause Low−Chronic:
Food consumption/gBW/day
Food(g)
0.20
0.15
0.10
0.05
0
50
100
Day
Treatment
Intact
VCD
VCD+E2
VCD+PBDE
VCD+E2+PBDE
VCD+E2+P4
VCD+E2+P4+PBDE
b
Food consumption/gBW/day
Perimenopause
High−Subacute
Menopause
High−Subacute
Menopause
Low−Chronic
Food_day_gBW
0.15
0.10
0.05
0.00
Intact
VCD
VCD+E2
VCD+PBDE
VCD+E2+PBDE
VCD+E2+P4
VCD+E2+P4+PBDE
c
Estimated total PBDE−47 exposure/gBW
Perimenopause
High−Subacute
Menopause
High−Subacute
Menopause
Low−Chronic
PBDE
0.008
0.006
0.004
0.002
0.000
VCD+PBDE
VCD+E2+PBDE
VCD+E2+P4+PBDE

Supplementary Figure 6 Food consumption and estimated peroral exposure to PBDE-47. **(A)** Food consumption (per gram body weight per day) of the animals in the Menopause Low-Chronic PBDE model. Day 0 indicates the start of the PBDE diet. **(B)** Averaged food consumptions (per gram body weight per day) during the PBDEs exposure in the three models. **(C)** Estimated total exposure (per gram body weight) to PBDE-47 during the experiments calculated from the food consumption.

a

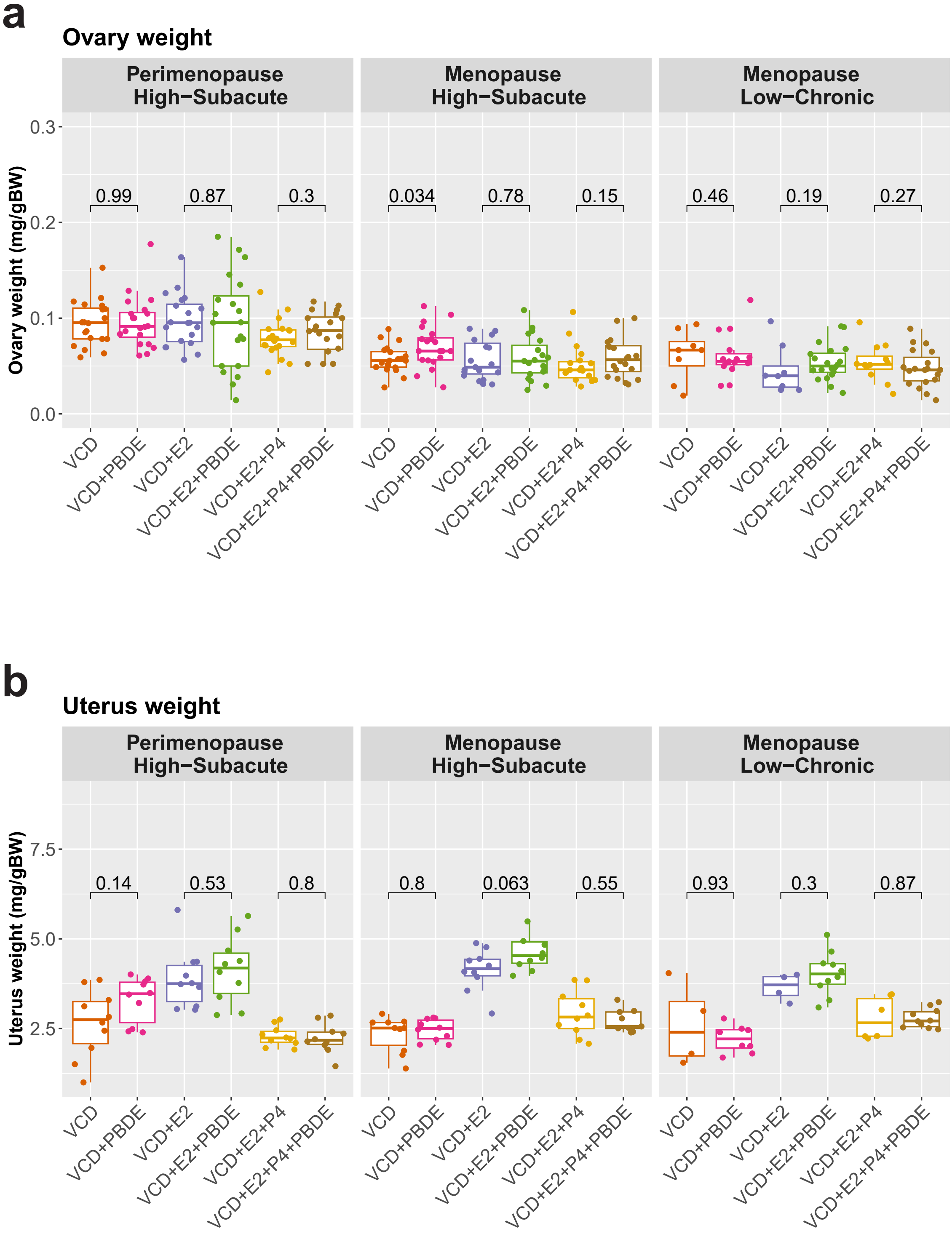
Ovary weight
Perimenopause High−Subacute
Menopause High−Subacute
Menopause Low−Chronic
Ovary weight (mg/gBW)
0.0
0.1
0.2
0.3
0.99
0.87
0.3
0.034
0.78
0.15
0.46
0.19
0.27
VCD
VCD+PBDE
VCD+E2
VCD+E2+PBDE
VCD+E2+P4
VCD+E2+P4+PBDE
Uterus weight
Perimenopause High−Subacute
Menopause High−Subacute
Menopause Low−Chronic
Uterus weight (mg/gBW)
2.5
5.0
7.5
0.14
0.53
0.8
0.8
0.063
0.55
0.93
0.3
0.87
VCD
VCD+PBDE
VCD+E2
VCD+E2+PBDE
VCD+E2+P4
VCD+E2+P4+PBDE

b

Supplementary Figure 7 Effects of the PBDEs exposure on the ovarian (A) and the uterine weight (B) in the three experiments. The box-plot elements were defined as follows: center line, median; box limits, upper and lower quartiles; whiskers, 1.5x interquartile range; points, outliers. The numbers above brackets indicate p-values.

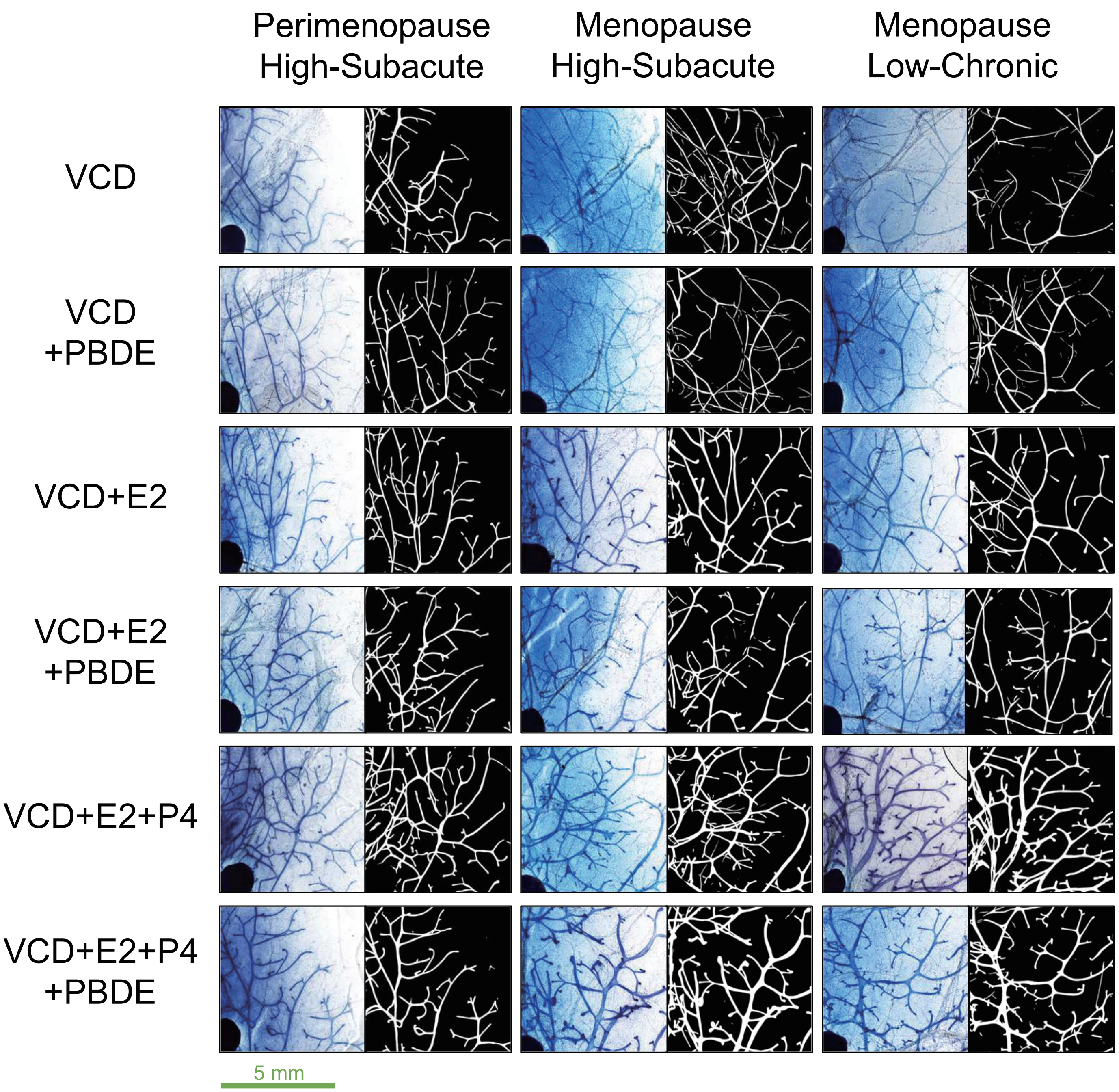
Perimenopause
High-Subacute
Menopause
High-Subacute
Menopause
Low-Chronic
VCD
VCD
+PBDE
VCD+E2
VCD+E2
+PBDE
VCD+E2+P4
VCD+E2+P4
+PBDE
5 mm

Supplementary Figure 8 Representative images of the mammary gland after the PBDEs exposure in the three models. A pair of images consist of the center area of the stained mammary gland with the lymph node positioned at the bottom left (left panel) and black-and-white segmentation of ductal structures of the identical area for the subsequent image analysis (right panel).

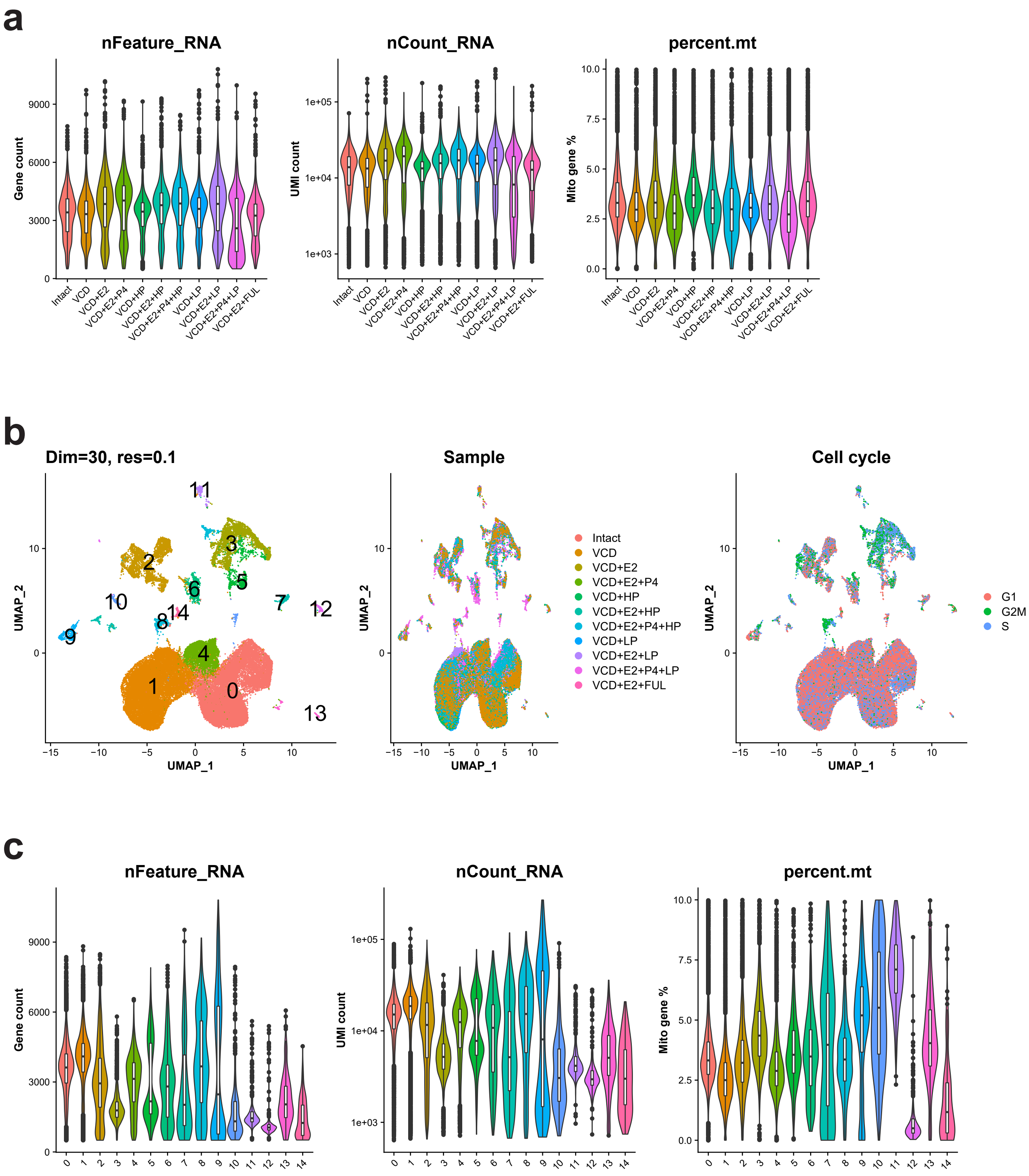
a
nFeature_RNA
nCount_RNA
percent.mt
Gene count
UMI count
Mito gene %
Intact
VCD
VCD+E2
VCD+E2+P4
VCD+HP
VCD+E2+HP
VCD+E2+P4+HP
VCD+LP
VCD+E2+LP
VCD+E2+P4+LP
VCD+E2+FUL
b
Dim=30, res=0.1
Sample
Cell cycle
UMAP_1
UMAP_2
G1
G2M
S
c
nFeature_RNA
nCount_RNA
percent.mt
Gene count
UMI count
Mito gene %

d

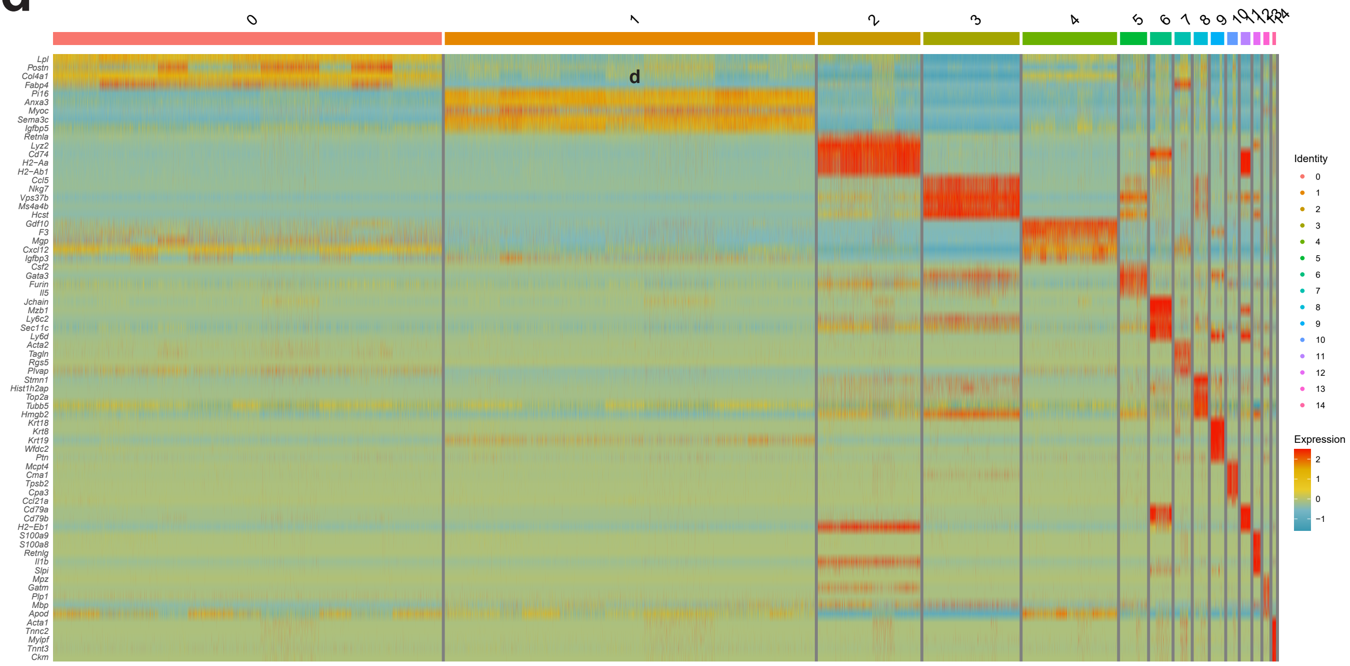
0
1
2
3
4
5
6
7
8
9
10
11
12
13
14
d
Lpl
Postn
Col4a1
Fabp4
Pi16
Anxa3
Myoc
Sema3c
Igfbp5
Retnla
Lyz2
Cd74
H2−Aa
H2−Ab1
Ccl5
Nkg7
Vps37b
Ms4a4b
Hcst
Gdf10
F3
Mgp
Cxcl12
Igfbp3
Csf2
Gata3
Furin
Il5
Jchain
Mzb1
Ly6c2
Sec11c
Ly6d
Acta2
Tagln
Rgs5
Plvap
Stmn1
Hist1h2ap
Top2a
Tubb5
Hmgb2
Krt18
Krt8
Krt19
Wfdc2
Ptn
Mcpt4
Cma1
Tpsb2
Cpa3
Ccl21a
Cd79a
Cd79b
H2−Eb1
S100a9
S100a8
Retnlg
Il1b
Slpi
Mpz
Gatm
Plp1
Mbp
Apod
Acta1
Tnnc2
Mylpf
Tnnt3
Ckm
Identity
Expression
2
1
0
−1

e

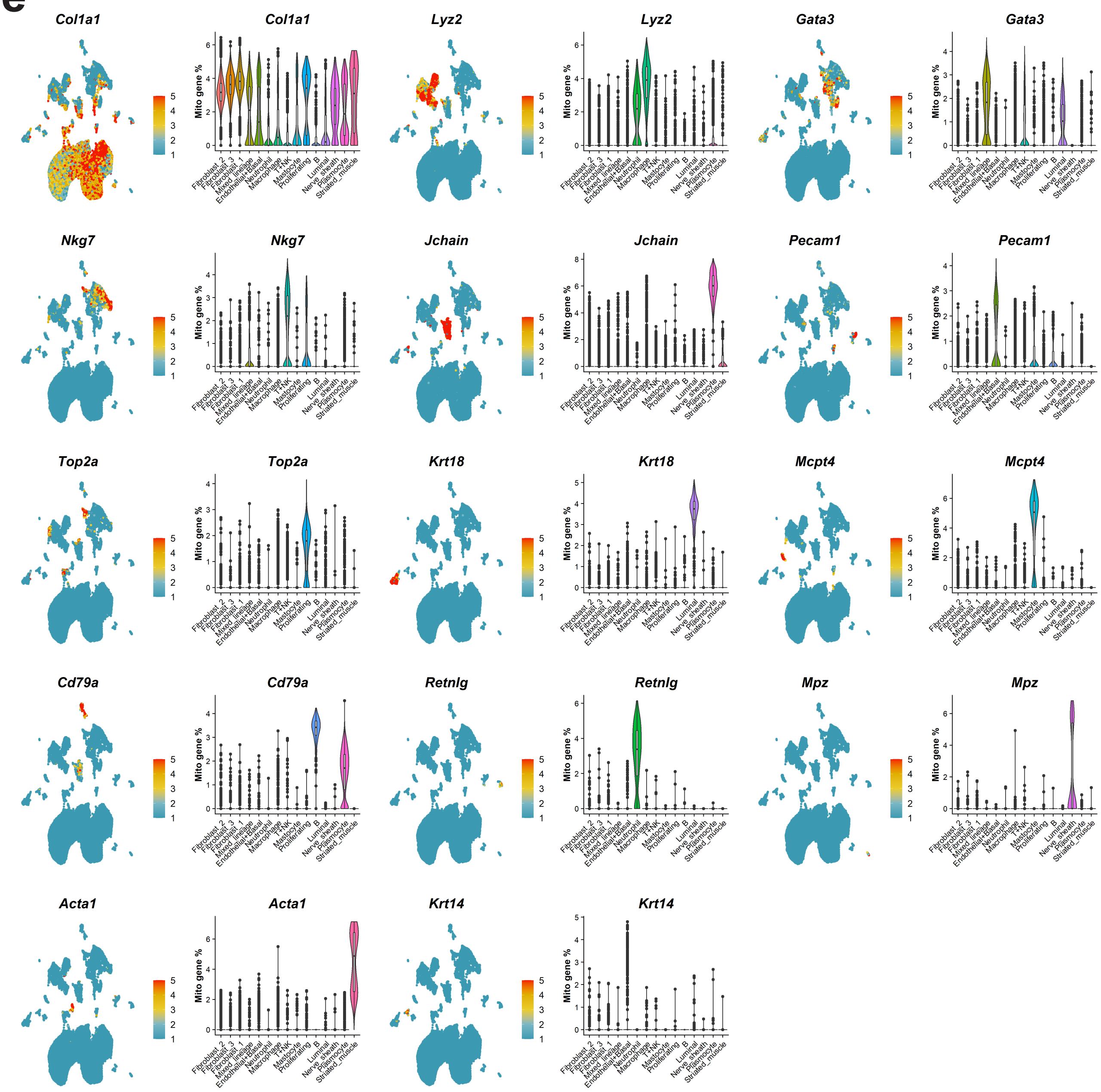
Col1a1
Lyz2
Gata3
Nkg7
Jchain
Pecam1
Top2a
Krt18
Mcpt4
Cd79a
Retnlg
Mpz
Acta1
Krt14
Mito gene %
Fibroblast_2
Fibroblast_3
Fibroblast_1
Mixed_lineage
Endothelial+Basal
Neutrophil
Macrophage
T+NK
Mastocyte
Proliferating
B
Luminal
Nerve_sheath
Plasmocyte
Striated_muscle

Supplementary Figure 9 The quality assessment and the preprocessing of the single-cell RNA sequencing data. (A) Distribution of the number of detected genes (nFeature_RNA) and transcripts (nCount_RNA) and the percentage of the mitochondrial genes (percent.mt) in each sample. **(B)** The 2D UMAP projections of the merged data color-coded by the clusters detected with the Louvain algorithm (left), the samples (middle), the cell cycle (right). **(C)** Distribution of the number of detected genes (nFeature_RNA) and transcripts (nCount_RNA) and the percentage of the mitochondrial genes (percent.mt) in each cluster. **(D)** The heatmap showing the expression of the top five genes in each cluster. **(E)** The expression of the marker genes for the individual cell types visualized on the UMAP plots (left panel) and the violin plots grouped by the clusters (right panel). *Col1a1;* fibroblasts, *Lyz2*; macrophages, *Gata3*; T cells, *Nkg7*; natural killer cells, *Jchain*; plasmocytes, *Pecam1*; endothelial cells, *Top2a*; proliferating cells, *Krt18*; luminal cells, *Mcpt4*; mast cells, *Cd79a*; B cells, Retnlg; Neutrophils, *Mpz*; nerve sheath cells, *Acta1*; striated muscle cells, *Krt14*; basal cells.

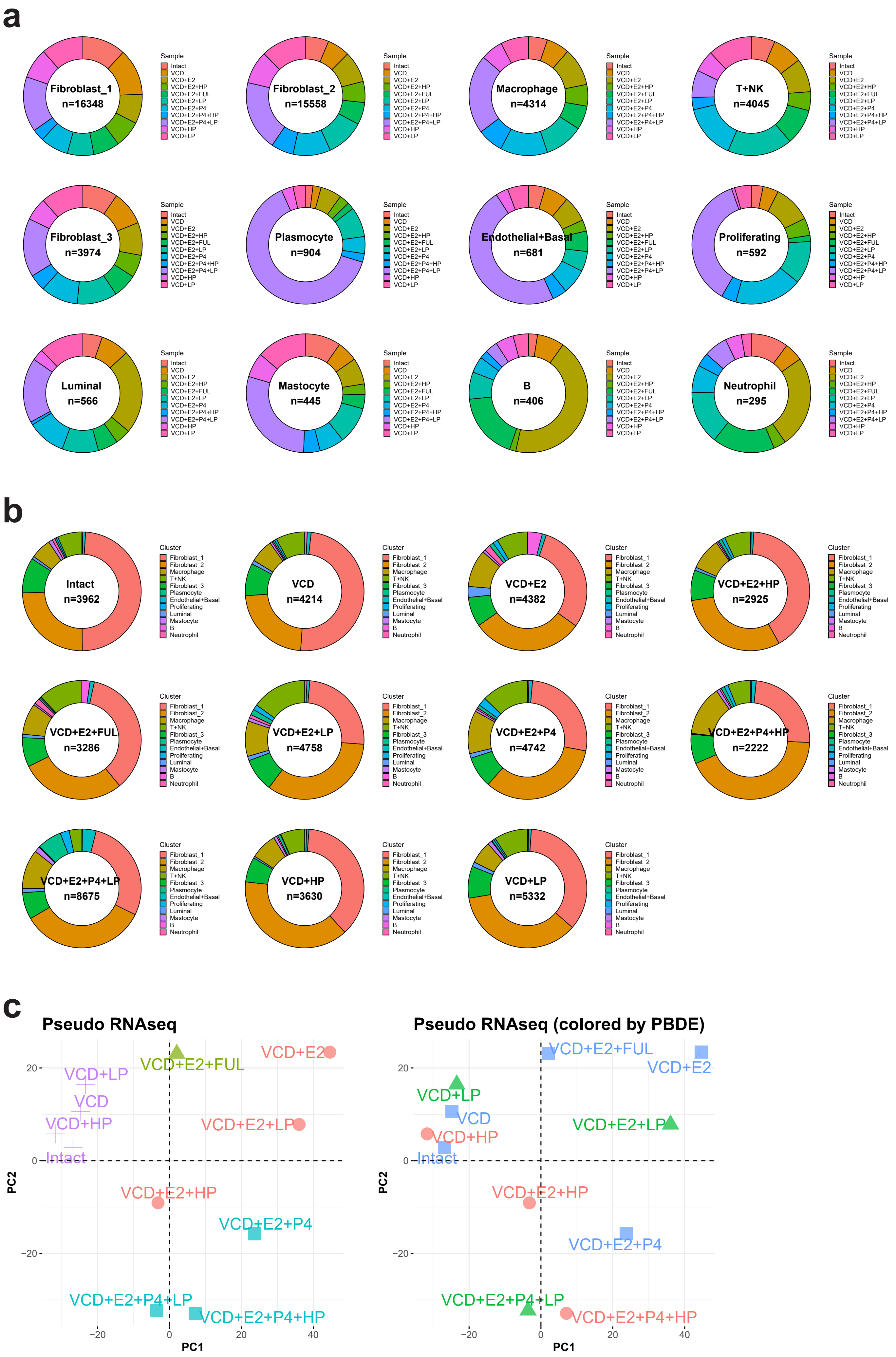
a
Fibroblast_1 n=16348
Fibroblast_2 n=15558
Macrophage n=4314
T+NK n=4045
Fibroblast_3 n=3974
Plasmocyte n=904
Endothelial+Basal n=681
Proliferating n=592
Luminal n=566
Mastocyte n=445
B n=406
Neutrophil n=295
Sample
Intact
VCD
VCD+E2
VCD+E2+HP
VCD+E2+FUL
VCD+E2+LP
VCD+E2+P4
VCD+E2+P4+HP
VCD+E2+P4+LP
VCD+HP
VCD+LP
b
Intact n=3962
VCD n=4214
VCD+E2 n=4382
VCD+E2+HP n=2925
VCD+E2+FUL n=3286
VCD+E2+LP n=4758
VCD+E2+P4 n=4742
VCD+E2+P4+HP n=2222
VCD+E2+P4+LP n=8675
VCD+HP n=3630
VCD+LP n=5332
Cluster
Fibroblast_1
Fibroblast_2
Macrophage
T+NK
Fibroblast_3
Plasmocyte
Endothelial+Basal
Proliferating
Luminal
Mastocyte
B
Neutrophil
c
Pseudo RNAseq
VCD+E2
VCD+E2+FUL
VCD+LP
VCD
VCD+HP
Intact
VCD+E2+LP
VCD+E2+HP
VCD+E2+P4
VCD+E2+P4+LP
VCD+E2+P4+HP
Pseudo RNAseq (colored by PBDE)
VCD+E2+FUL
VCD+E2
VCD+LP
VCD
VCD+HP
Intact
VCD+E2+LP
VCD+E2+HP
VCD+E2+P4
VCD+E2+P4+LP
VCD+E2+P4+HP
PC2
PC1
20
0
-20
-20
0
20
40

Supplementary Figure 10 The breakdowns and the principal component analysis of the single-cell RNA sequencing data. (A) The breakdowns of each cluster by the samples. **(B)** The breakdowns of each sample by the clusters. **(C)** The principal component analysis of the pseudo RNA seq data color-coded by the hormone replacement treatments (left, purple; none, red; E2, green; E2+FUL, blue; E2+P4) and the PBDE treatments (right, blue; none, green; low PBDEs, blue; high PBDEs).

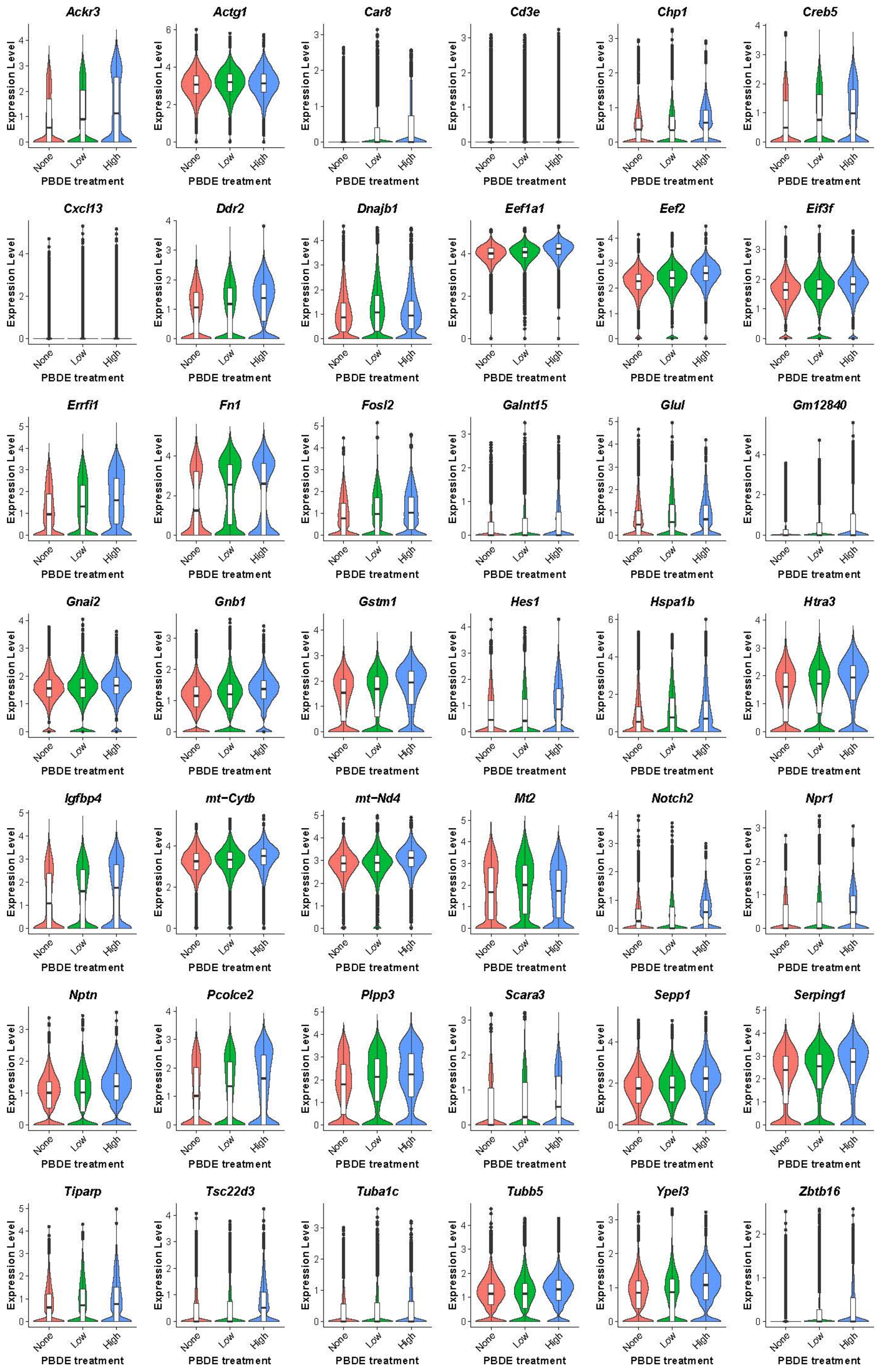
Ackr3
Actg1
Car8
Cd3e
Chp1
Creb5
Cxcl13
Ddr2
Dnajb1
Eef1a1
Eef2
Eif3f
Errfi1
Fn1
Fosl2
Galnt15
Glul
Gm12840
Gnai2
Gnb1
Gstm1
Hes1
Hspa1b
Htra3
Igfbp4
mt−Cytb
mt−Nd4
Mt2
Notch2
Npr1
Nptn
Pcolce2
Plpp3
Scara3
Sepp1
Serping1
Tiparp
Tsc22d3
Tuba1c
Tubb5
Ypel3
Zbtb16
Expression Level
PBDE treatment
None
Low
High

Supplementary Figure 11 The expression of the genes with significant upregulation in the Low-Chronic or the High-Subacute PBDEs groups. The expression of a gene of interest is visualized on the violin plot stratified by the PBDEs exposure. The None group; VCD, VCD+E2, and VCD+E2+P4. The Low group; VCD+LP, VCD+E2+LP, and VCD+E2+P4+LP. The High group; VCD+HP, VCD+E2+HP, and VCD+E2+P4+HP.

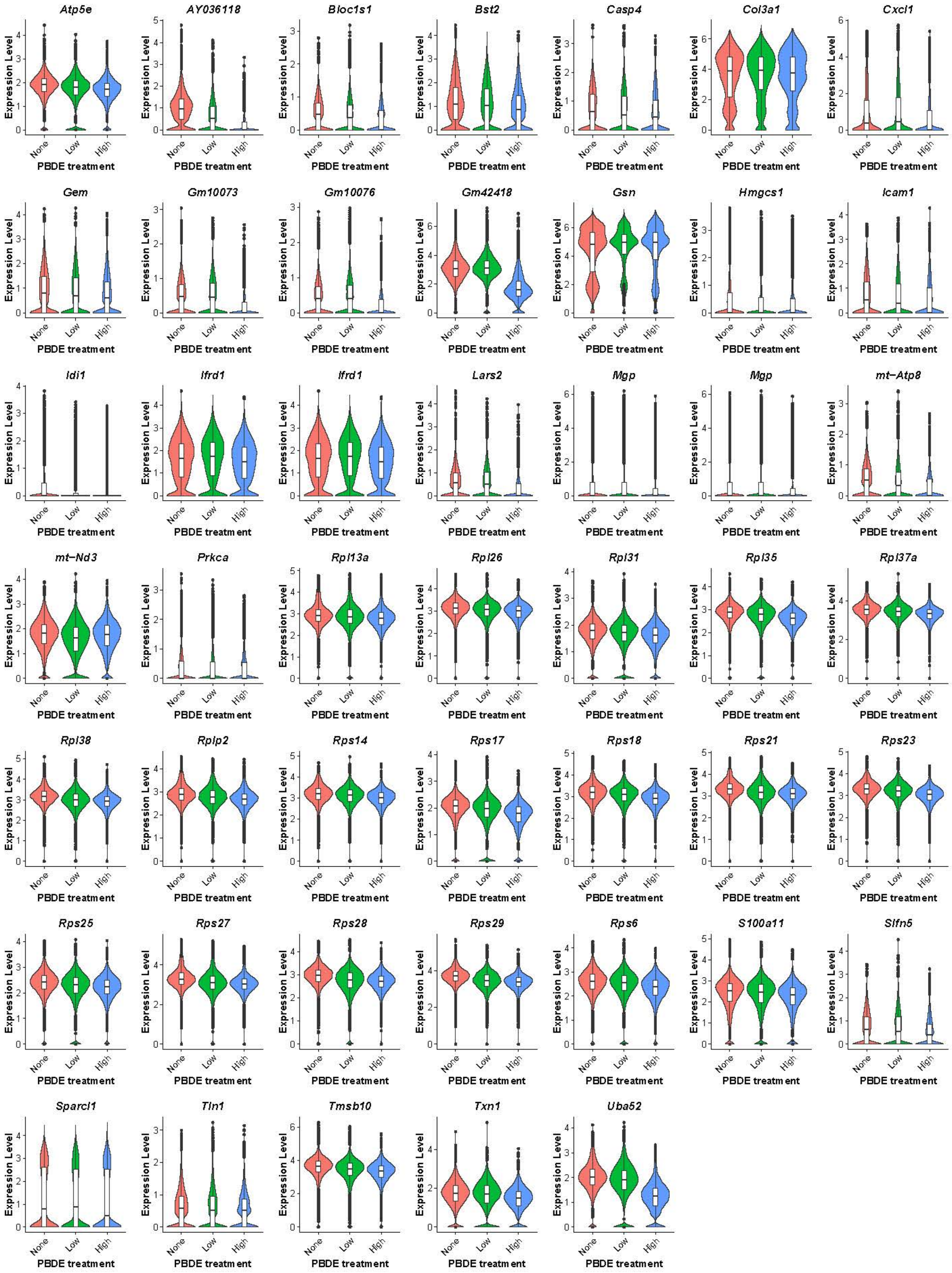
Atp5e
AY036118
Bloc1s1
Bst2
Casp4
Col3a1
Cxcl1
Gem
Gm10073
Gm10076
Gm42418
Gsn
Hmgcs1
Icam1
Idi1
Ifrd1
Ifrd1
Lars2
Mgp
Mgp
mt−Atp8
mt−Nd3
Prkca
Rpl13a
Rpl26
Rpl31
Rpl35
Rpl37a
Rpl38
Rplp2
Rps14
Rps17
Rps18
Rps21
Rps23
Rps25
Rps27
Rps28
Rps29
Rps6
S100a11
Slfn5
Sparcl1
Tln1
Tmsb10
Txn1
Uba52
Expression Level
PBDE treatment
None
Low
High

Supplementary Figure 12 The expression of the genes with significant downregulation in the Low-Chronic or the High-Subacute PBDEs groups. The expression of a gene of interest is visualized on the violin plot stratified by the PBDEs exposure. The None group; VCD, VCD+E2, and VCD+E2+P4. The Low group; VCD+LP, VCD+E2+LP, and VCD+E2+P4+LP. The High group; VCD+HP, VCD+E2+HP, and VCD+E2+P4+HP.

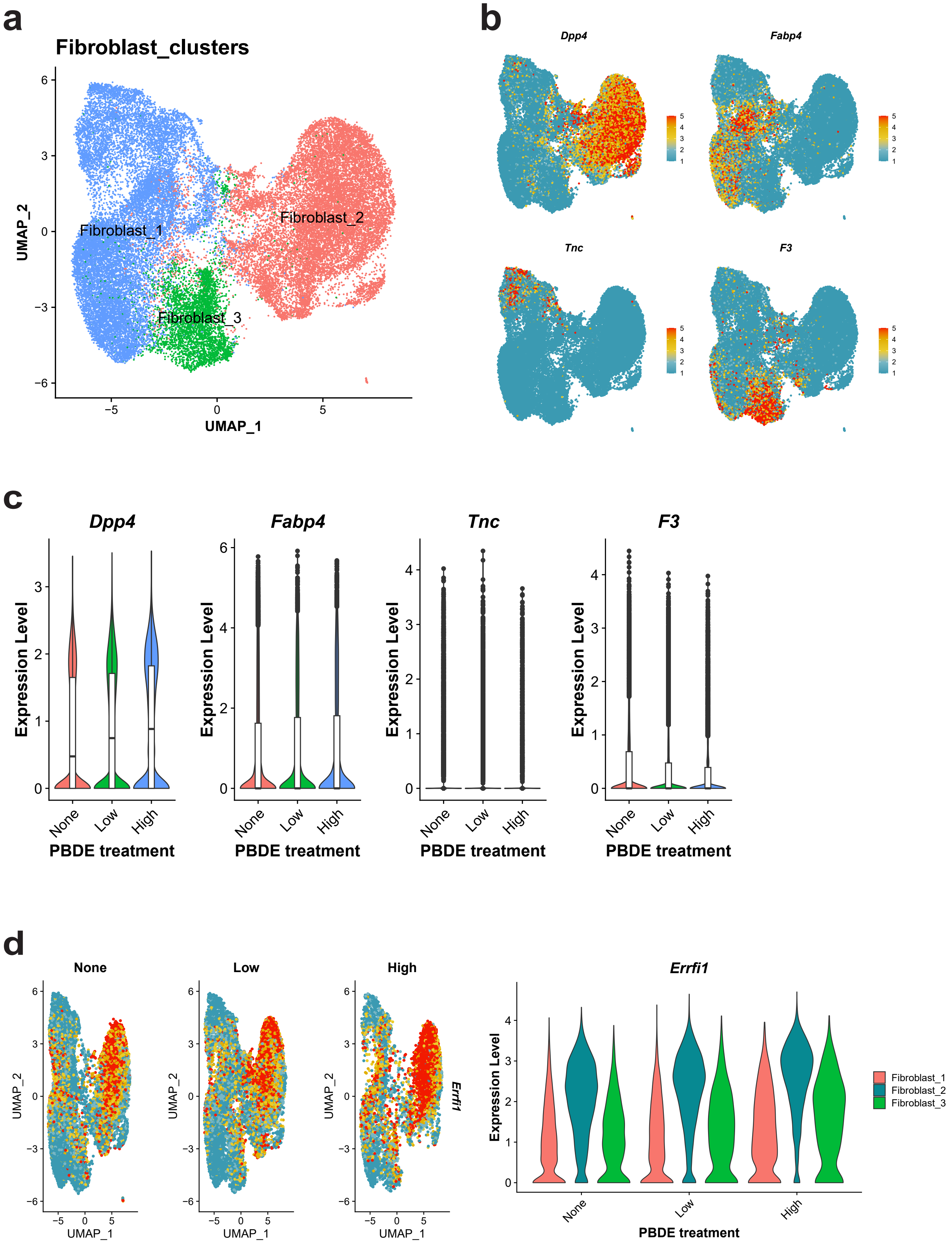

a
Fibroblast_clusters
Fibroblast_1
Fibroblast_2
Fibroblast_3
UMAP_1
UMAP_2
b
Dpp4
Fabp4
Tnc
F3
c
Dpp4
Fabp4
Tnc
F3
Expression Level
None
Low
High
PBDE treatment
d
None
Low
High
UMAP_1
UMAP_2
Errfi1
Errfi1
Expression Level
PBDE treatment
Fibroblast_1
Fibroblast_2
Fibroblast_3

Supplementary Figure 13 Sub analysis in the fibrobrast populations. (A) Fibroblast reclustering on a UMAP plot. **(B)** Expression of the marker genes for “progenitor” (*Dpp4*), “adipogenic” (*Fabp4*), “ECM” (*Tnc*) and “adipo-regulatory” (*F3*) fibroblasts on the UMAP plots. **(C)** Expression of the marker genes on the violin plot stratified by the PBDEs exposure. **(D)** Expression of *Errfi1* on the UMAP (left) and the violin (right) plot stratified by the PBDEs exposure and the clusters. The None group; VCD, VCD+E2, and VCD+E2+P4. The Low group; VCD+LP, VCD+E2+LP, and VCD+E2+P4+LP. The High group; VCD+HP, VCD+E2+HP, and VCD+E2+P4+HP.

References

1. Kanaya N, Chang G, Wu X, Saeki K, Bernal L, Shim H-J, et al. Single-cell RNA-sequencing analysis of estrogen- and endocrine-disrupting chemical-induced reorganization of mouse mammary gland. Communications Biology. 2019;2:1–15.

2. Saeki K, Chang G, Kanaya N, Wu X, Wang J, Bernal L, et al. Mammary cell gene expression atlas links epithelial cell remodeling events to breast carcinogenesis. Commun Biol. 2021;4:1–16.

3. Hurley S, Goldberg D, Nelson DO, Guo W, Wang Y, Baek H-G, et al. Temporal Evaluation of Polybrominated Diphenyl Ether (PBDE) Serum Levels in Middle-Aged and Older California Women, 2011-2015. Environ Sci Technol. 2017;51:4697–704.
